# Long ties, disruptive life events, and economic prosperity

**DOI:** 10.1073/pnas.2211062120

**Published:** 2023-07-06

**Authors:** Eaman Jahani, Samuel P. Fraiberger, Michael Bailey, Dean Eckles

**Affiliations:** ^a^Sloan School of Management, Massachusetts Institute of Technology, Cambridge, MA 02142; ^b^Institute for Data, Systems, and Society, Massachusetts Institute of Technology, Cambridge, MA 02142; ^c^Department of Statistics, University of California, Berkeley, CA 94720; ^d^Development Impact Evaluation, World Bank, Washington, DC 20433; ^e^Meta, Computational Social Science Team, Menlo Park, CA 94025

**Keywords:** social networks, weak ties, structural diversity, economic outcomes

## Abstract

Social networks shape and reflect economic life. Prior studies have identified long ties, which connect people who lack mutual contacts, as a correlate of individuals’ success within firms and places’ economic prosperity. However, we lack population-scale evidence of the individual-level link between long ties and economic prosperity, and why some people have more long ties remains obscure. Here, using a social network constructed from interactions on Facebook, we establish a robust association between long ties and economic outcomes and study disruptive life events hypothesized to cause formation of long ties. Consistent with prior aggregated results, administrative units with a higher fraction of long ties tend to have higher-income and economic mobility. Individuals with more long ties live in higher-income places and have higher values of proxies for economic prosperity (e.g., using more Internet-connected devices and making more donations). Furthermore, having stronger long ties (i.e., with higher intensity of interaction) is associated with better outcomes, consistent with an advantage from the structural diversity constituted by long ties, rather than them being weak ties per se. We then study the role of disruptive life events in the formation of long ties. Individuals who have migrated between US states, have transferred between high schools, or have attended college out-of-state have a higher fraction of long ties among their contacts many years after the event. Overall, these results suggest that long ties are robustly associated with economic prosperity and highlight roles for important life experiences in developing and maintaining long ties.

## Significance

Long ties, which connect individuals who lack mutual contacts in a social network, are known to be important for accessing valuable information about economic opportunities. Using population-scale communication data, we provide evidence of a robust positive relationship between the share of long ties and economic outcomes both at the individual and local levels in the United States and Mexico. Furthermore, we uncover that the formation of long ties is associated with disruptive life events such as migrating to another state or transferring to a different high school, which points to potential mechanisms responsible for forming and maintaining these valuable connections.

Social networks can shape access to information and opportunities (1–4) and, thus, economic prosperity for individuals and places. Likewise, economic activity can affect the formation and maintenance of social ties. Such reciprocal causal relationships can produce robust and even self-reinforcing associations between network structure and economic prosperity, both for individuals and geographic regions. Indeed, recent empirical work relying on digital interaction data at a population-wide scale has documented some notable associations between the characteristics of social connections in a region and economic outcomes in that region. In particular, places with a higher structural and geographic diversity of connections have better economic outcomes (5–8). For example, the relative frequency of Facebook friendship links between every county-pair in the United States is a strong predictor of socioeconomic characteristics such as income, education, teenage birth rate, or life expectancy at the county level (8).

These aggregate associations between network structure and economic propensity are consistent with a broader theoretical and empirical literature on network structure. Long ties, which connect individuals who do not share any mutual contact (i.e., local bridges, ties with embeddedness of 0), are thought to facilitate the circulation of novel and valuable information and behaviors between multiple communities (9–14). Structurally diverse networks, which comprise numerous long ties, can provide individuals with an information advantage which leads to better managerial performance (11, 15–18), a higher propensity to find jobs (19, 20), more innovative teams (21–23), and faster adoption of new technologies (13, 24–26). Despite the extensive literature arguing for benefits of structural diversity (i.e., having a network with more long ties) in specialized professional contexts, the relationship between such network structures and general measures of economic prosperity is underexplored. Population-scale evidence has largely relied on ecological regressions with network structures and economic outcomes aggregated to telecommunication (6) or administrative regions (8), and association with network structure has often been characterized with univariate correlations and/or by combining multiple measures of network structure into a composite index (6). Furthermore, the question of who has more long ties and why they form and maintain these ties is largely unexplored despite their importance.

We have two main contributions: establishing a robust association between long ties and important measures of economic prosperity and linking major, disruptive life events to formation of long ties. First, we present population-scale evidence on the relationship between structural diversity and socioeconomic outcomes. Individual Facebook users in the United States with more long ties have better outcomes along four proxies of socioeconomic well-being, including living in richer zip codes, having more internet-connected devices, more expensive phones, and donating more often to charitable fundraising campaigns. Furthermore, we examine the additional role of tie strength, which captures the intensity of interactions between individuals. Individuals with stronger long ties tend to have better economic outcomes, even after accounting for the prevalence of these ties and their total intensity of interactions. Both of these results are also reflected in geographically aggregated results in the United States and Mexico. Structural diversity appears to importantly characterize the networks of higher-income people and places.

Given the robust association between long ties and economic outcomes, we then probe some potential origins in specific life events. Few studies have focused on the determinants of long ties. Existing works have shown that brokers, who connect two otherwise disconnected communities by filling a structural hole, are less risk-averse, more entrepreneurial, and more prone to change (10, 27, 28). They have a higher ability to integrate with various social groups and form structurally diverse networks arguably because they are less likely to define themselves and others in terms of socially distinct categories (29, 30). While these studies suggest that personality traits are associated with the presence of long ties, personality traits are affected by life experience (31) and network measures related to long ties have large nonheritable components (32, 33). We therefore study life events that expose individuals to multiple communities and push them to establish connections with individuals who might be very different from their previous contacts: i) migrating to another state, ii) transferring between high schools, and iii) attending an out-of-state college. In all three cases, experiencing such disruptions is associated with having more structurally diverse networks years later, even excluding the ties most directly implicated (e.g., ties to alumni of the involved schools). Though we flexibly adjust for covariates, we cannot attribute these associations entirely to causal effects of these events; however, in the case of transferring between high schools, a quasi-experimental analysis focusing on school closures yields qualitatively similar results. Together these results provide a view of long ties as consistently linked to disruptive life events, even long after those events.

## Long Ties and Economic Outcomes

How does structural diversity relate to economic outcomes? To answer this question, we first count all the public comments posted on Facebook between December 2020 and June 2021 between users living in the United States to form a network of interactions between users; this is the network used throughout the paper (*Materials and Methods* for details). As a first measure of structural diversity, we compute the fraction of each individual’s ties that are long ties (i.e., lack any mutual network neighbors). We begin with a brief presentation of geographically aggregated results in the United States and Mexico, before highlighting our main results at the individual level in the United States.

**Fig. 1.**
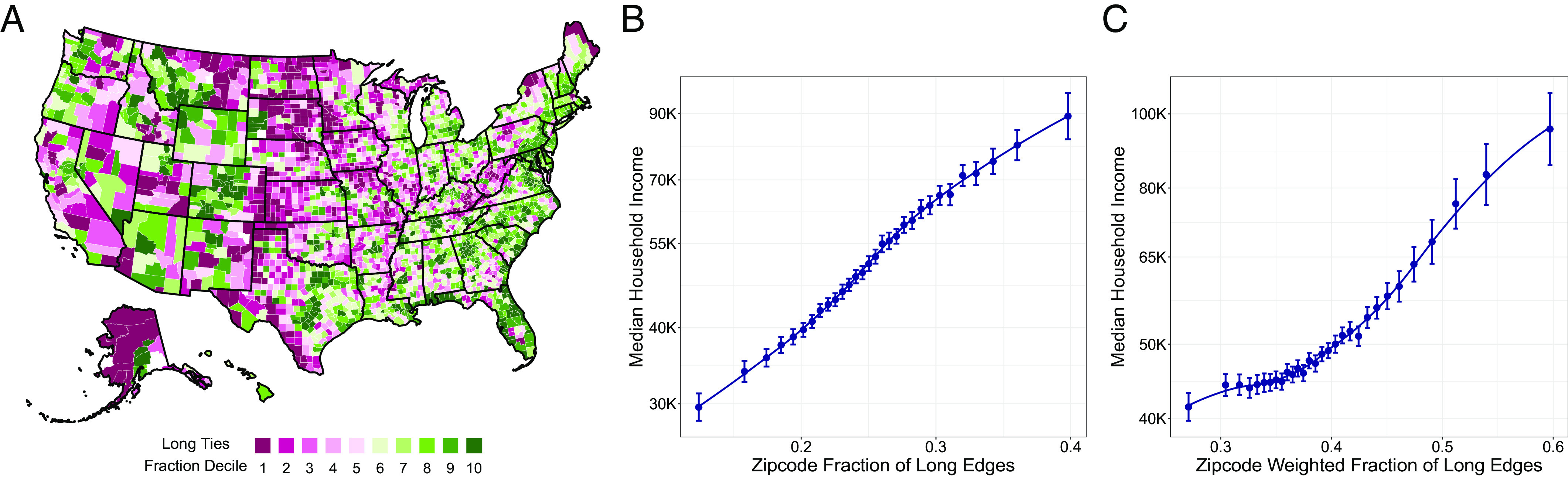
(*A*) Fraction of long ties, measured across counties in the United States, after controlling for average degree. The adjusted (residualized) fraction of long ties across administrative units in each country is binned into 10 deciles, with lower values shown in purple and higher values shown in green. Administrative units with less than 200 Facebook users sufficiently active over the measurement period or with less than 500 individuals are shown in white. (*B*) The fraction of long ties and (*C*) the tie-strength weighted fraction of long ties predict median income in US zip codes. The binned regression plots are generated according to models (**6**) and (**7**) in *Materials and Methods*, which adjust for the number of nodes in the zip code, its racial composition, and average degree; the regression with weighted fraction of long ties also adjusts for the fraction of long ties and the sum of the weights. Solid lines are local smoothers of second degree; bars are 95% confidence intervals, which are cluster-robust at the county level.

Pooling the networks of all Facebook users living in the same region provides an overview of variation in this measure of network structure (Fig. 1*A* and *SI Appendix*, Fig. S3*A*). The least structurally diverse regions with available data, such as Appalachia in the United States or Zacatecas in Mexico, are economically disadvantaged. Zip codes in the United States with more long ties have higher household incomes (Fig. 1*B*), even after flexibly controlling for their overall population and racial composition, the number of Facebook users residing in the zip code, and their average degree (*Materials and Methods* for details). This positive relationship holds for additional measures of economic prosperity in the United States, including social mobility and unemployment rates, and for a wealth index in Mexico (*SI Appendix*, Fig. S4).

**Fig. 2.**
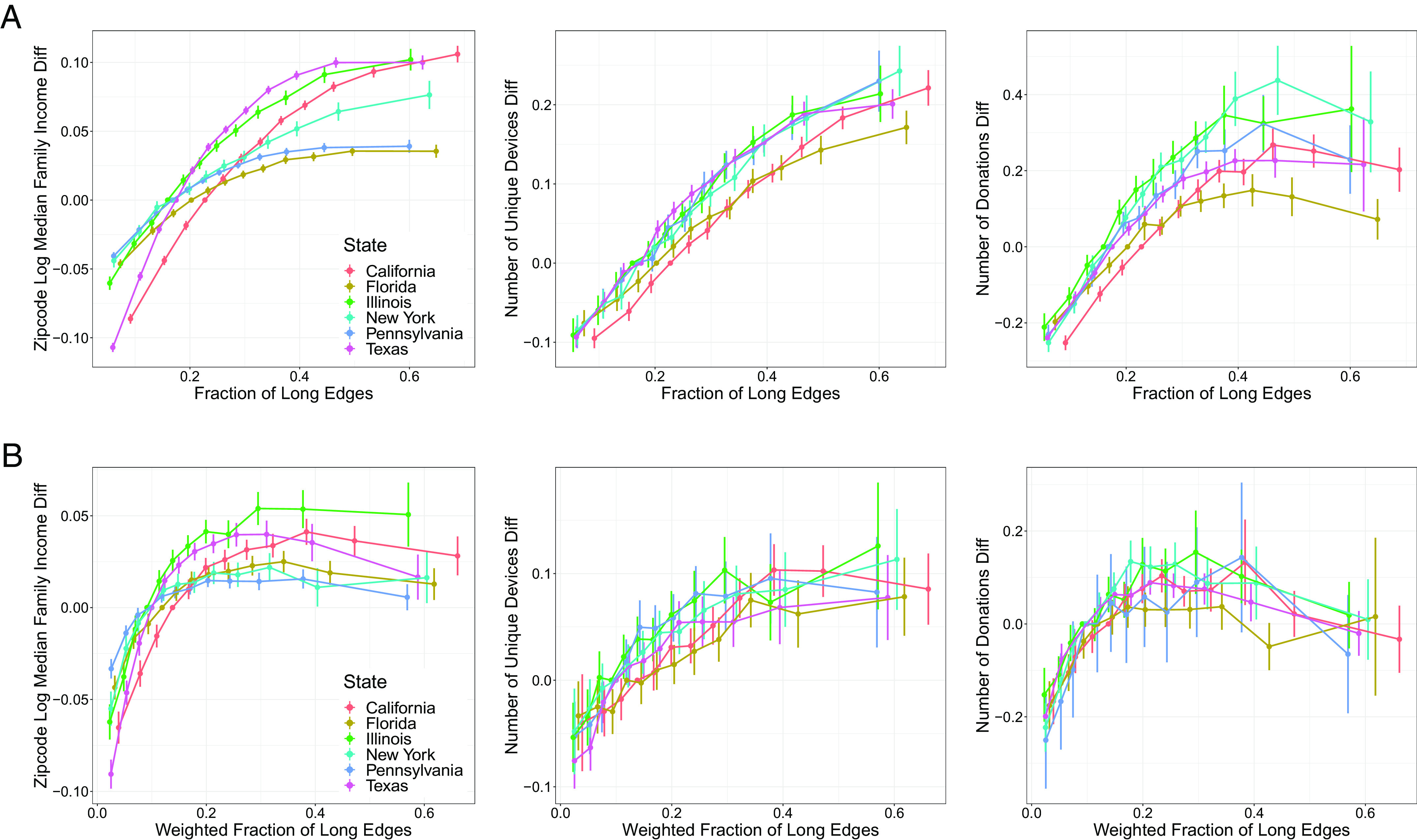
(A) Fraction of long ties and (B) tie strength weighted fraction of long ties in ego networks versus individual-level variables that are positively correlated with proxies for economic outcomes for users who reside in the *Top* 6 most populous states in the United States. Users are matched on degree, average degree of their contacts, daily login activity, average daily login activity of their contacts, age, and gender; see *Materials and Methods*. The matching for the bottom row additionally incorporates the fraction of long ties and average edge weight. Values in the y-axis correspond to the difference with the baseline represented with a value of zero. Bars are 95% confidence intervals for these differences assuming independent observations; robustness to within-state dependence is illustrated by the multiple state-specific results.

The findings from ecological regressions such as the analysis above do not necessarily imply a specific individual-level association. Thus, our primary analyses examine associations at the level of individuals’, rather than places’, networks. As typical measures of economic outcomes (e.g., income, wealth) are not available at an individual level linked to Facebook users, we rely on proxy measures of economic prosperity (34). For individuals living in the six most populous US states, we divide the fraction and weighted fraction of long ties each into 12 equal-sized categories and match on degree, level of daily activity, gender, and age (*Materials and Methods* for details). Individuals with a higher fraction of long ties live in zip codes with higher median family income, use more distinct devices to access Facebook, and donate more often to fundraising campaigns on Facebook (Fig. 2), consistent with previous findings that richer people donate more often (35). They similarly use more expensive mobile phones and have visited more countries in short trips (*SI Appendix*, Fig. S6).

By using a network weighted by the number of interactions, we examine the interplay between structural diversity and tie strength. Weak ties, which connect people who infrequently interact with one another, are often described as providing access to more novel—and thus incrementally valuable—information than strong ones (9). However, while weak ties tend to be long ties in general, there is substantial variation, and even some very long ties spanning distant parts of a network can be strong (36). This motivates an explicit distinction between tie strength and tie length (10, 18, 37). So, as a second measure of structural diversity, in addition to the fraction of long ties, we compute a tie-strength weighted fraction of long ties; *Materials and Methods* for details. When comparing individuals with a similar fraction of long ties, those with stronger long ties (i.e., higher values of weighted fraction of long ties) live in higher-income zip codes, use more unique devices, and make more donations (Fig. 2*B*). Similar results hold for additional proxies (*SI Appendix*, Fig. S6*B*), and at a geographically aggregated level for median income in the United States (Fig. 1*C*) and other economic outcomes in the United States and Mexico (*SI Appendix*, Fig. S4).

**Fig. 3.**
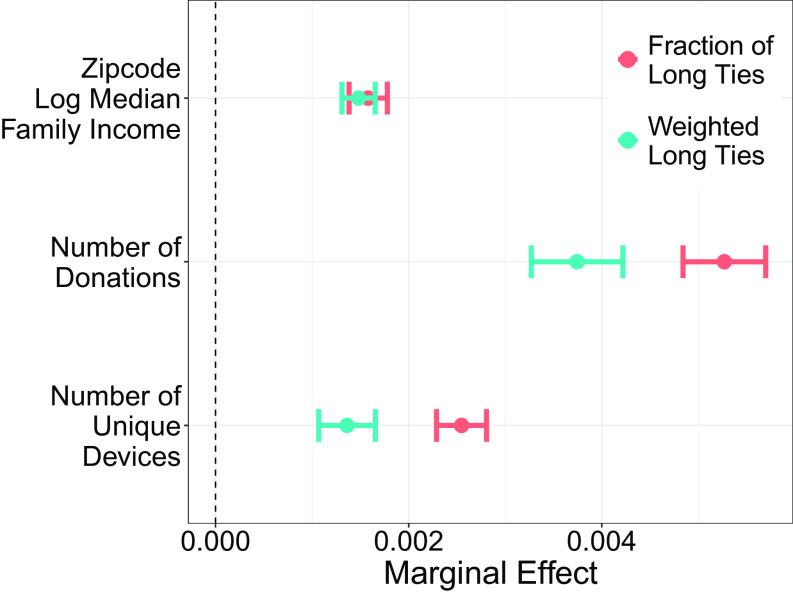
Estimated average marginal change in measures of individual economic prosperity given a 1% point (0.01) increase in the fraction or weighted fraction of long ties. These are computed from random forest models fit to each state and DC and including degree, average degree of contacts, daily login activity of the user, average daily login activity of their contacts, average weight of user’s edges, age, and gender. Bars are 95% confidence intervals that are cluster-robust at the state level.

To jointly examine the contribution of the fraction and weighted fraction of long ties, we fit random forests (38) that use these fractions and other covariates to predict each of the economic outcomes. The average marginal change in the predicted outcomes from increasing the fraction and weighted fraction of long ties are positive and are of similar magnitude to each other (Fig. 3) where averages are computed over US states, covariates, and the distribution of the long tie measures. These results are quite consistent in direction when considering US states individually (*SI Appendix*, Fig. S7) suggesting that having long and strong ties provides a robust signal for predicting economic prosperity.

Taken together, we find evidence that people and places with more long ties and stronger long ties are more economically prosperous. Having stronger long ties, rather than weak ties per se, predicts economic prosperity.

## Life Events and Long Ties

Having established the existence of a robust cross-sectional relationship between the prevalence of long ties and economic prosperity, we turn our focus to processes responsible for their formation. We study three life events, each involving mobility between institutions and/or places, that we hypothesized would enable individuals to build connections to disparate communities: migrating to a new state, attending an out-of-state college, and transferring to a different high school. In all three cases, we compare the structural diversity of the network—many years later—surrounding individuals who experienced such events with those who did not.

### Interstate Migration.

**Fig. 4.**
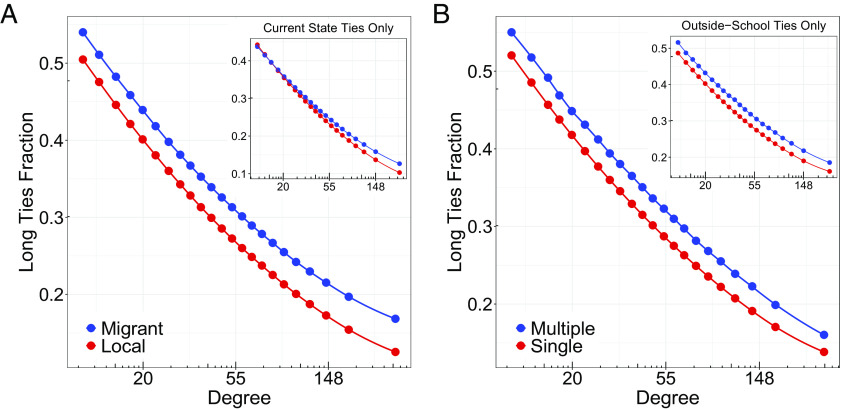
Conditional on degree, people who (*A*) are interstate migrants or (*B*) attended multiple high schools have more long ties than matched controls. Qualitatively similar relationships hold when restricting the analysis to less directly implicated ties—those within the current state (*A inset*) and those outside of high school (*B inset*). All estimates are poststratified by gender, age, and hometown county income bins.

To study interstate migration and structural diversity, we examine Facebook users born in the United States and who have lived in the same state since 2012. We compare the fraction of long ties for individuals who were born in a different state from the one they currently live to that for individuals living in the state where they were born, matching on degree, age, gender, and home county income (*Materials and Methods* for details). Interstate migrants have on average 13% more long ties relative to nonmigrants (33.7% vs. 29.7% in absolute percentage terms of all their contacts with difference-in-means *P* <  10^−10^ using cluster-robust standard errors at the county level). While this difference is in part driven by migrants having accumulated long ties in their previous state, we also find that migrants have 4.0% more long ties with residents of their current state relative to nonmigrants (28.7% vs. 27.6% in absolute percentage terms with difference-in-means *P* = 0.011 using county-level cluster-robust standard errors). To account for differences in ego network sizes, we compare migrants’ propensity to form long ties with that of nonmigrants who have a similar degree. Migrants consistently have more long ties than nonmigrants with a similar degree (Fig. 4*A*). This difference in structural diversity between migrants and nonmigrants increases with the number of contacts and persists when we consider only long ties to contacts living in their current state. Thus, interstate migrants form long ties at a higher rate than similar nonmigrants independently of their number of contacts, and even in the state where they currently live.

### Out-of-State College.

To analyze attending an out-of-state college or university, we study Facebook users born in the United States and who attended college outside of their home state, and compare them with users of the same cohort who attended college within their home state. Attending an out-of-state college is associated with having a higher fraction of long ties years after attending college, and the effect persists even when we focus on connections to friends made outside of college and when we match on whether they are currently interstate migrants (*SI Appendix*, Fig. S12). See *SI Appendix*, section D.4 for more detailed analysis of this life event.

### Transferring High Schools.

Adolescence involves learning how to expand one’s social network (39), and therefore disruptive experiences during this period are likely to shape an individual’s network structure later in life (40). We focus on Facebook users who were born between 1961 and 1991 in the United States, went to high school, and are currently living in the United States. We then compare the ego networks of users who report attending multiple high schools within the same state with those who attended a single high school (thus ruling out the effect of interstate relocation), while matching on degree, age, gender, and income of their home county ( *Materials and Methods* for details). People who transferred to a different high school on average have 10% more long ties relative to those who attended a single high school (35.2% vs. 31.9% in absolute percentage terms of all their contacts with difference-in-means *P* <  10^−10^ using cluster-robust standard errors at the county level). Some of this difference could be due to an increase in the immediate opportunity of meeting new friends. We therefore conduct an analysis restricted to long ties formed with contacts outside of high school, finding a fraction of long ties for users who transferred to a different high school that remains 9% larger relative to those who did not transfer (34.8% vs. 31.8% in absolute terms with *P* <  10^−10^ using county-level cluster-robust standard errors). Users who attended multiple high schools consistently form more long ties across all values of the ego network degree (Fig. 4*B*), even when we consider only contacts outside of high school.

**Fig. 5.**
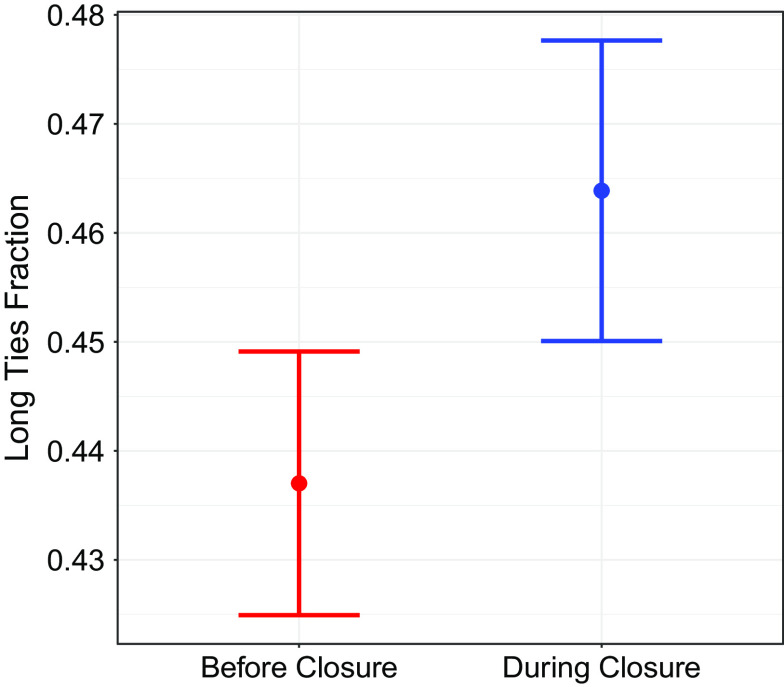
People who attended a high school during its closure have more long ties than those who attended the same high school prior to closure. The estimates are poststratified by school and age. Bars are bootstrap 95% confidence intervals that are cluster-robust at the school level.

Even conditional on covariates, any causal effects of transferring high schools (like the other two life events) on network structure are likely confounded with other unobserved variables. Here, we consider some plausibly exogenous variation in transferring high schools by identifying high schools that closed prior to 2016. Among attendees of these high schools around the time of the closure, later cohorts report that they have attended multiple high schools 20% more relative to earlier cohorts (17.3% vs 14.3% in absolute percentage terms); both fractions are high, likely reflecting various factors affecting transfers prior to outright closure. Within this restricted sample, and matching on school and age, we find people in the closure-affected cohorts have 6.1% more long ties relative to before-closure cohorts (46.4% vs. 43.7% in absolute percentage terms, Fig. 5). In a linear model with fixed effects for school and age, we estimate that being in a closure-affected cohort increases the fraction of long ties by 1.2 percentage points (95% CI [0.4, 1.9], *SI Appendix*, Table S1). Similar results hold when considering only the ties made outside high school. Together, these findings suggest that transferring to a different high school shapes the proclivity to connect with different communities.

Finally, in *SI Appendix*, section D.6 we provide some evidence that individuals having each of these life events above is also associated with better economic outcomes measured with the same proxy variables as in prior analyses (*SI Appendix*, Figs. S14, S15, and S16).

## Discussion

There is an extensive literature on the role played by social network structures in constituting social capital (2, 34, 41–43). A long tie is potentially a source of information not as directly available through any other contact, therefore individuals with structurally diverse networks ought to have an informational advantage (10, 11, 15–18, 27). Our study documents a strong cross-sectional relationship between long ties and measures of economic prosperity at both geographically aggregated and individual levels. The literature has sometimes equated weak ties and long ties, for example, by treating the number of mutual contacts as a measure of tie strength (44–46), such that long ties are all counted as “weak”. Our measure of tie strength allows us to clarify that it is long ties and stronger long ties (rather than weak ties per se) that are particularly associated with prosperity, consistent with the mechanisms posited by both Granovetter (9) and subsequent work (10, 18). Our results suggest that tie strength and length should be treated as conceptually distinct dimensions, each with its own links to economic prosperity.

Prompted by this link between long ties and economic outcomes, we consider the origins of long ties and factors that enable people to create and maintain these ties. Seminal work by Simmel (47) suggests that individuals with long ties are mediators who can resolve potential conflicts between disconnected communities due to their unique involvement with both communities. Simmel discussed the ability of these mediators as a natural consequence of their structural position in the network; however, it is perceivable that certain individuals have a higher tendency to occupy these network positions (*SI Appendix*, Fig S8). The existing literature on the origins of long ties has characterized the personality of individuals with long ties to be entrepreneurial, risk-taking, and thriving on change (28, 29, 48).

The studies mentioned above treat the determinants of long ties as a matter of personality or even genetic heritability (32), rather than a skill or disposition to be acquired, even though many of the personality correlates of long ties can develop through experiences. We examine the link between experiencing major life disruptions—interstate migration, transferring high schools and attending an out-of-state college—and the formation of these ties. The experiences from these major disruptions are similar in the sense that they expose individuals to diverse communities, require them to form new ties with others who are different than their old network, and potentially cause development of the social skills to do so. We find that individuals who experienced such disruptions have more structurally diverse networks years later, in ways that are not mechanically due to geographic mobility or exposure to a new community during high school or college. These findings suggest that the tendency to form long ties is not only a matter of innate personality but also life experiences. Shaping one’s personality and acquiring the necessary social skills is one potential mechanism behind the effect of these disruptive life experiences.

These results are consistent with findings showing that the benefits of long ties come from *oscillation* between making deep investments and brokering in multiple communities (49). Brokers with network oscillation have developed the skill for successfully moving in and out of groups, much similar to the experience of migration or relocation during high school:

“Experience with change is preparation for change... [Oscillators] can be expected to be flexible in moving between identities ... and so develop the adaptive self-monitoring associated with network brokers. The image that comes to mind is people who grew up in multiple countries, or in families that frequently moved between cities.” (49, p. 387]

Our findings have important limitations. First, we use communication activity on Facebook as a measure of social networks more generally (i.e., interaction offline and via other media). Our choice for network edges, i.e., reciprocal interactions, is meant to limit the network to meaningful ties; nevertheless, it is possible that our network measurements do not accurately capture off-Facebook interactions that confer economic advantage. Second, while the analyses of network structure and economic outcomes flexibly adjust for or match on relevant covariates, some of the observed associations can be due to unobserved factors. Furthermore, the observed associations likely reflect causal processes in both directions; that is, social networks affect economic outcomes, and also economic prosperity affects network formation and maintenance. For example, wealthier people may be more able to form and maintain long ties (by, e.g., traveling, dining out). Similarly, selection into some professions can lead to more opportunities to form and maintain long ties. There may also be important feedback between processes in these two directions if wealth enables forming long ties, which are then advantageous economically, etc. Our observational data cannot unambiguously disentangle these mechanisms. Overall, our results fit with the growing literature on benefits of long ties in specific settings and outcomes, and some of these studies rely on randomized interventions that affect network structure (46). We regard both causal directions as of interest for further study.

While the three disruptive life events we studied were selected because we hypothesized they would increase structural diversity, there can also be confounding factors between groups here. However, having three different types of events—involving different processes by which people are selected into the disruption—pointing to the same conclusions makes for a more robust and notable pattern. In addition, as we examine the network interactions of an individual many years after experiencing a disruptive event, we rule out endogeneities occurring on a short time scale. Finally, we argue that transferring high schools, especially when due to high school closure, is the most plausibly exogenous event. Transferring high schools is often either a decision made by parents on behalf of students or results from external factors outside a student’s control (50–52). It may even result from their current school closing, which we also found is associated with forming more long ties. Taken together, we believe that our findings on the role of disruptive life events are unlikely to be fully attributed to confounding.

Understanding the origins and consequences of long ties can have important implications for public policy. Intergenerational economic mobility has been declining in the United States (53), but long ties (*SI Appendix*, Figs. S4 and S5) and ties spanning class boundaries are associated with higher economic mobility (34). Some of those interclass ties are likely attributable to the design of institutions, as, e.g., smaller high schools have more such ties (43). More generally, we posit that dispositions to form long ties—developed in part through the kind of disruptive life events studied here—are upstream of many ties that cut across groups, including interclass ties. Policies aimed at fostering structural diversity could have a positive impact on social mobility and economic prosperity more generally. For these reasons, we hope that this study will encourage further research into the mechanisms of formation of long ties, particularly as it pertains to skills that can be taught, and their causal links with economic prosperity.

## Materials and Methods

First, we describe the data and methods for examining the link between long ties and economic outcomes at the individual level, with additional notes for the geographically aggregated level. We then explain the data collection and analysis of the determinants of long ties.

### Individual-Level Long Ties and Economic Outcomes.

#### Ego networks.

For each user currently residing in the United States, we construct their ego network from public commenting communication data spanning a period of 6 mo from December 2020 to June 2021. An ego network includes all the edges between the user (the ego) and their contacts (alters) and the edges among contacts themselves. In the commenting network, there is a directed edge between two users if one has mentioned or replied to a post made by the other. In constructing the ego networks, we retain only reciprocal edges (with at least one comment exchanged in each direction) to exclude one-off interactions between individuals who would be otherwise unrelated. Furthermore, nonreciprocal edges have been shown to be less important in diffusion and peer influence processes in networks (54, 55). To ensure a minimal level of activity by each user, we discard those with degree less than 10 (have communicated with less than 10 people over the 6-mo period). Commenting activities are more appropriate for the construction of the network than the actual friendships on Facebook for two reasons: commenting contacts are more likely to be strong and relevant to each user, as opposed to many friends on Facebook who are only acquaintances without any meaningful interaction over a long period. But more importantly, Facebook users extensively use commenting as a form of communication as there are about 195 million daily active users on the Facebook website or its mobile application in the United States and Canada.^*^

#### Measuring long ties.

Structural diversity is a purely topological concept that designates a multiplicity of local communities, but which has been operationalized in multiple ways, even within a single study (13). The fraction of long ties, those without any mutual contacts, is a conceptually simple and computationally practical operationalization. Long ties are sometimes also called local bridges and are those ties that have an edge embeddedness of 0 (56, §3.3]. Long ties closely resemble the widely used concept of structural holes or social bridges and their overall prevalence may indicate the extent of novel information received.

The local clustering coefficient is a related measure to the fraction of long ties and has been widely used as a node-level measure of embeddedness (56). While the clustering coefficient aggregates edge embeddedness, the fraction of long ties would be a more appropriate measure for the frequency of completely unembedded ties. Thus, our focus is on the fraction of long ties; however, we reached similar conclusions using the clustering coefficient.

Given an ego network, we compute the fraction of long ties among all ties from the ego to their contacts:

[1] $$\begin{array}{c} l_{i}=\frac{\sum_{j\in N_{i}}I\left(\;{\text{mc}}_{\mathrm{ij}}=0\right)}{|N_{i}|}, \end{array}$$

where *N*_*i*_ is the set of *i*’s neighbors, *I*(.) is the indicator function, and mc_*ij*_ is the number of mutual contacts between *i* and *j*.

We also consider what fraction of total interaction by an individual occurs along long ties. The normalized count of exchanged comments is our measure of (normalized) tie strength:

[2] $$\begin{array}{c} w_{\mathrm{ij}}=\frac{t_{\mathrm{ij}}}{t_{i}}, \end{array}$$

where *t*_*ij*_ is the number of comments *i* has exchanged with *j* in the 6 mo measurement period and *t*_*i*_ = ∑_*j* ∈ *N*_*i*__*t*_*ij*_ is the total number of comments user *i* has exchanged with all their contacts. Similar definitions of tie strength are widely used [e.g., refs. 57 and 58], and are known to correlate with other survey-based measures of tie strength (59, 60).

Given this definition of tie strength, a simple and intuitive measure for the strength of long ties is their weighted sum. The analysis on the strength of long ties uses the weighted fraction of long ties in which each long tie is weighted by its normalized tie strength defined in Eq. **2**:

[3] $$\begin{array}{c} l_{i}^{w}=\sum_{j\in N_{i}}w_{ij}I(\;{\text{mc}}_{ij}=0). \end{array}$$

Both *l*_*i*_ and *l*_*i*_^*w*^ can range from 0 to 1.

#### Economic measures.

Direct measures of economic well-being are not available at the individual level linked to Facebook users. However, various proxy measures that are related to economic outcomes are available at the individual level. Our analysis relies on the following three proxy outcome variables in the main text:

1. The median and mean household income of the user’s residential zip code: The income value is obtained from 2018 ACS and the user zip code of residence is based on a prediction that incorporates various pieces of information including internet connection. Note that this is not strictly an individual-level outcome.
2. Unique devices: A user connection to Facebook logs their device model. We count the number of unique devices a user has used more than once to connect to Facebook from 2019 to 2021.
3. Number of donations to fundraisers: Facebook has a personal fundraising platform where each user can request for financial assistance either for personal reasons or to a charity organization. Since 2016 when fundraisers launched to 2021, about $5 billion dollars have been donated through Facebook fundraising platform, involving about 85 million people.^†^ We count the number of times a user has made a donation to a fundraising campaign over a 2-y period from June 2019 to June 2021. We expect that users with a higher disposable income tend to make more donations.

In *SI Appendix*, we provide further evidence of this relationship by examining the following two proxy outcomes:

1. The user’s phone price: A user connection to Facebook logs their mobile phone model. We match their phone model against a database of phone prices in dollars.
2. The user’s international touristic trips: The number of countries, other than Canada and Mexico, visited by a user in trips shorter than two weeks since 2012 captures the user’s tourism activity. A visit to a country is inferred based on the connection IP of the user while on a trip.

#### Matching and estimation.

We compare individuals with different values of the focal structural diversity measures but similar values of other covariates. We discretize the fraction (or weighted) long ties into 12 equal-sized levels. Generalized full matching (61) is an appropriate matching framework here as it allows for more than two treatment arms (12 in our case), assigns all units to exactly one matching group, and is computationally tractable. Fig. 2 shows the difference between each level of fraction of long ties or weighted long ties and the fourth bin which is used as the base level. The matching is done once per each state, rather than the whole country, which uses only the data from users whose predicted current residence is in that state. This allows us to discover potential heterogeneities that might exist between the states.

The Euclidean distance metric in the matching procedure is studentized so that different variables with different ranges contribute equally to the distance metric. It is constructed based on the following variables when the independent variable is the fraction of long ties (*Top row* of Fig. 2):

1. Log user degree and log average degree of their contacts. These two variables ensure comparisons are made between ego networks of similar sizes.
2. Daily login activity of the user, measured in terms of the number of days over a month with a login, and the average daily login activity of their contacts. These two variables ensure that the ego and alters have similar levels of activity, which might affect the fraction of long ties in the ego network.
3. Age and gender of the user.

When the independent variable is weighted long ties (bottom row of Fig. 2), the distance metric incorporates the following additional variables:

1. Log average weight of user’s edges ($\log(\frac{t_{i}}{|N_{i}|})$). This ensures that matched users have similar levels of commenting activity since, as this affects the feasible ranges of weighted long ties *l*_*i*_^*w*^
2. Fraction of long ties *l*_*i*_.

#### Predictive model.

*SI Appendix*, Section B.1 in reports on a different estimation approach based on the average marginal effect of fraction of or weighted long ties in a random forest model. Fig. 3 reports summaries of these models fit to data from each state. The results of this alternative procedure are consistent with the matching results presented above.

### Geographically Aggregated Long Ties and Economic Outcomes.

#### Data on network interactions.

These analyses use the same communication network as in the individual-level analyses. For each Facebook user, we also observe the predicted city of residence, which relies on the user’s information and activity, for example, their self-reported city on their profile and their internet connection information. These residential predictions allow us to match users to zip codes, counties, and states and construct the corresponding zip code network, consisting of users residing in the zip code.

#### Construction of geographically aggregated networks.

The nodes in each zip code network consist of all users who reside in that zip code and all their contacts. (Throughout we focus on zip codes, though the maps in Fig. 1*A* and *SI Appendix*, Fig. S3 use aggregation to counties with otherwise identical logic.) As with the individual-level analyses, we consider only reciprocal ties to filter out one-off communication events that do not signify a meaningful relationship. Thus, the existence of an edge in the zip code network means there has been at least one communication event in each direction. As opposed to the individual-level results which used directed networks, edges in the aggregate zip code networks are undirected with weights that correspond to the total number of communication events in either direction from December 2020 to June 2021. *SI Appendix*, Fig. S1 shows a schematic view of the zip code networks. In our analysis, we consider only zip codes with at least 150 users who reside in that zip code and for which we have census data available. This leaves us with 26,429 zip codes in the United States (excluding PO Boxes or businesses) and 15,157 zip codes in Mexico for which we have both network and economic indicator data. *SI Appendix*, Fig. S2 shows the histogram of some basic characteristics of these networks in the United States, along with a few economic indicators.

#### Measuring long ties in geographically aggregated networks.

We use the fraction of long ties among all edges that involve at least one node inside the zip code as our primary measure of zip code structural diversity:

[4] $$\begin{array}{c} l_{z}=\frac{\sum_{i\in Z}\sum_{j\in N_{i}}I\left(\;{\text{mc}}_{\mathrm{ij}}=0\right)}{\sum_{i\in Z}\left|N_{i}\right|}, \end{array}$$

where *Z* is the set of nodes residing inside the zip code. These long ties are shown with dotted black lines in *SI Appendix*, Fig. S1.

As with the individual-level analysis, we also define a version of this measure weighted by tie strength:

[5] $$\begin{array}{c} l_{z}^{w}=\frac{\sum_{i\in Z}\sum_{j\in N_{i}}w_{\mathrm{ij}}I\left(\;{\text{mc}}_{\mathrm{ij}}=0\right)}{\left|Z\right|}. \end{array}$$

Since the measure of tie strength is normalized, *l*_*z*_^*w*^ ranges between 0 and 1 and can be treated as *weighted fraction of long ties*. *SI Appendix*, Figs. S4 and S5 compare both *l*_*z*_ and *l*_*z*_^*w*^ measures against zip code outcomes.

#### Data on economic indicators.

In the United States, the zip code outcome variables consist of a set of economic indicators obtained from the census 5-year estimates of the 2018 American Community Survey (ACS) at the level of zip code tabulation areas (ZCTA). The economic indicators we consider here are median household income, fraction of households with income below $25K, and the unemployment rate. We also consider measures of social mobility provided by the Atlas of Opportunity project at the ZCTA level (62):

1. the probability of a child born in the zip code and from the bottom 25% of the income distribution in the United States reaching the top 20% of the income distribution and
2. the percentile income rank of a child born in the zip code from the bottom 25% of the income distribution.

Finally, as a robustness check, we replicate these analyses in Mexico. Due to the lack of income data at the zip code level, we constructed a wealth index for each zip code using the first principal component of the following variables from the 2020 census: percentage of illiterate population aged 15 or more, population aged 6 to 14 not attending school, population aged 15 or more without complete elementary school, population with no access to health services, log of average occupants per room, and percentage of households with dirt floor, no toilet, no piped water, no sewer system, no electricity, no washing machine, or no fridge. The wealth index for each zip code is the value of the first component rescaled to between 0 and 1. Effectively, our outcome indicator in Mexico is a summary measure on the intensity of economic, health, and education prosperity.

#### Covariates.

There is variation in the size of zip code networks in ways that can influence the range of reasonable values for *l*_*z*_ and affect its statistical relationship with the outcomes. Thus, we control for the following variables in our analysis: population of the zip code, number of network nodes residing in the zip code, and mean number of edges from a node inside the zip code (mean degree). All the economic indicators we consider are correlated with racial composition of the zip code. Thus, to ensure that our network measure explains any outcome variation above and beyond racial composition, we also control for the racial composition (percent white) in each zip code. The data from Mexico do not include zip code-level racial composition, so the model for Mexico simply controls for the network covariates mentioned above.

Our second goal is to establish the relationship between the strength of long ties and the economic outcomes given a fixed number of long ties. Since *l*_*z*_^*w*^ is highly correlated with the unweighted measure of structural diversity, *l*_*z*_, our analysis must control for it. Thus, our second analysis controls for the fraction of long ties, *l*_*w*_, in addition to those mentioned above.

#### Model and estimation.

To enable discovering potential nonlinearity in relationships between the zip code outcome and network structure, we use a modern binscatter method, binsreg (63). In addition to visualizing the data with cluster-robust confidence intervals at each bin, binscatter provides a principled way to adjust for our control variables such that it does not suffer from problems of residual-based approaches (64).

Binscatter regression with covariate adjustment fits the data according to a linear model on the covariates. In order to avoid any assumption on the functional form of the covariates, they are binned and the model includes an indicator for each bin. For example, the model on the relationship between the zip code outcome and the fraction of long ties is:

[6] $$\begin{array}{c} \begin{array}{c} y_{z}=\mu \left(l_{z}\right)+\alpha p_{z}+\beta n_{z}d_{z}+\theta r_{z}+\epsilon_{z}\\ E[\epsilon_{z}|l_{z},p_{z},n_{z},d_{z},r_{z}]=0, \end{array} \end{array}$$

where *y*_*z*_ is an outcome measure (e.g., zip code mean household income), *l*_*z*_ is the fraction of long ties, ***p***_*z*_ is the zip code population bin, ***n***_*z*_ is the binned number of nodes inside the zip code, ***d***_*z*_ is the binned average degree of nodes inside the zip code, and ***r***_*z*_ is the percent white in the zip codes. The model attempts to make comparisons between networks of similar sizes by controlling for the combination of *n*_*z*_ and *d*_*z*_, such that *d*_*z*_ bins are determined conditional or within the *n*_*z*_ bin. Binscatter regression reveals the shape of function *μ*(.) and computes the standard errors of each bin. Overall the model above includes 87 indicators as covariates.

We employ a similar approach for the model on the relationship between zip code outcomes and the strength of long ties, with the additional controls for the fraction of long ties as formulated below.

[7] $$\begin{array}{c} \begin{array}{c} y_{z}=\mu^{\prime }\left(l_{z}^{w}\right)+\delta^{\prime }l_{z}+\alpha^{\prime }p_{z}+\beta^{\prime }n_{z}d_{z}+\theta^{\prime }r_{z}+\epsilon_{z}^{\prime }\\ E[\epsilon_{z}^{\prime }|l_{z}^{w},l_{z},p_{z},n_{z},d_{z},r_{z}]=0, \end{array} \end{array}$$

where *l*_*z*_^*w*^ is the weighted fraction of long ties, ***l***_*z*_ is the binned unweighted fraction of long ties. The model above includes 94 indicators as covariates.

### Life Events and Long Ties.

#### Data on ego networks.

The same network described in the previous sections is used to measure the fraction of long ties.

#### Data on interstate migration.

The current state of each user can be inferred based on Facebook’s internal prediction of residential location (based on activity and self-reported location information on their profile) as also used above in construction of zip-code-level networks. Given these data, we construct a monthly panel of predicted state location for each user for 9 y starting from January 2012. To determine whether a user has experienced interstate migration, we also need information on their hometown state, for which we rely on the optionally listed hometown on the user’s Facebook profile and ensure it can be matched against a known location. Overall, 72% of the active users in the data have provided this self-reported information on their profile. Given the monthly residence and hometown states, we restrict our attention to users with a hometown city in the United States, aged between 30 and 60 who have not migrated since 2012 (i.e., their monthly residence state has remained the same since January 2012). We then divide these users to migrants or those with a different hometown state than their current state and nonmigrants with the same current and hometown states. Therefore in our analysis, migrants are users who moved to their current state prior to 2012 and have lived in their current state continuously since 2012. Similarly, nonmigrants are users who have continued residing in their home state since 2012. The final data consist of 20.6 million users, out of which 16.7 million users (81%) are local or nonmigrant and 3.9 million users (19%) are migrant.

#### Data on high school attendance.

We obtain high school attendance data on each user based on the self-reported high school names and pages on their profiles. Multiple pages referring to the same high school by different users are consolidated to a single high school and then matched against the database of high schools in the United States provided by the National Center for Education Statistics (NCES) to ensure the validity of these self-reported schools. About 65% of the users we examined have reported their high school information. Our analysis is based on users aged between 30 and 60 who currently live in the United States, have indicated their hometown city in the United States, and have attended their high schools in the United States and all within a single state. We divide this population of users into two groups of single or multiple high schools and join each user with their ego network constructed from communication data. The final population consists of 24.4 million users, out of which 23.8 million (97%) attended a single high school and 673 thousand (3%) attended multiple high schools all within a single state.

#### Analysis.

Our goal is to compare the average fraction of long ties over the population of two groups: migrants vs. locals or multiple vs. single high school attendees. A difference in means within each degree bin, however, does not account for differences between the two populations. In our analysis, we account for observable differences using poststratification (i.e., many-to-many matching). In our poststratification, the combination of binned age (3 bins), gender (2 bins), and hometown county income (15 bins) constitute a stratum (a total of 90 strata). Fig. 4*A* and *B*, and *SI Appendix*, Fig. S12 show these poststratified means within each degree bin (with a total of 30 equal-size degree bins). We perform the poststratification conditional on degree for two reasons: first, to show how the range of common values for the fraction of long ties varies by degree and, second, to show that the difference between two groups (migrants vs. locals or single vs. multiple high schools attendance) is consistent across the whole range of ego degrees.

While migrant and local populations have similar degree and gender distributions, they vary significantly by their age, mean household income of where they grew up, and current state (*SI Appendix*, Fig. S9), hence the need for stratification. Similarly, users who have attended multiple high schools tend to have a slightly higher degree and be of younger age than the single high school users (*SI Appendix*, Fig. S10). Our analysis does not include migration status in the strata definition for two reasons: First, as we see in *SI Appendix*, Fig. S10, there is no notable difference between the two high school groups in terms of migration. Second, our analysis requires that neither group experiences an interstate migration event during high school.

### High School Closures and Long Ties.

#### Data.

The National Council for Education Statistics provides data on high schools that closed in each year from 1995 to 2016. The analysis on high school closures involves Facebook users who had reported attending any of these closed high schools and have provided the exact time period of their attendance. The users whose reported end date at the school is within one year of its closure are considered to be in the “During Closure” condition and those whose reported end date at the school is between 2 and 5 y prior to its closure constitute the “Before Closure” condition. The final data consist of 33,813 users who had attended 285 closed high schools, out of which 11,309 (22,504) users attended the high schools during (before) their closure.

#### Analysis.

The main result reported above is based on *SI Appendix*, Table S1 which uses a two-way fixed effect model with fixed effects for both age in whole years and schools and cluster-robust standard errors at the school level. The average fractions of long ties in Fig. 5 are estimated using poststratification with the combination of school and age in 4-y bins as the strata.

## Supplementary Material

Appendix 01 (PDF)

## Data Availability

Anonymized replication materials (code and sufficient statistics for all analyses in the paper) have been deposited in Github (https://github.com/eamanj/long_ties_economic_outcomes) (65). In addition, the publication of this paper is accompanied by the public release of geographically-aggregated measures of network structure as used in Fig. 1 and other associated analyses (https://socialmediaarchive.org/record/27). In order to protect the privacy of individuals and comply with relevant regulations, this data release has used methods that ensure the aggregated data does not reveal much about any individual person.

## Footnotes

^*^These numbers are from a recent earnings report: https://investor.fb.com/financials/default.aspx.

^†^www.facebook.com/business/news/coming-together-to-raise-5-billion-on-facebook-and-instagram-.
