# Supplementary material for: Long ties, disruptive life events, and economic prosperity
Source: Proc Natl Acad Sci U S A. 2023 Jul 6;120(28):e2211062120. doi: 10.1073/pnas.2211062120 (PMC10334764; doi:10.1073/pnas.2211062120)
Supplement: Supplementary file 1 — Appendix 01 (PDF) [file pnas.2211062120.sapp.pdf]

Supplementary Information for “Long ties, disruptive life events and economic prosperity”

Contents

A Geographically-Aggregated Associations 2

B Individual-level Associations 3
B.1 Average Marginal Effects with Random Forests . . . 4

C Demographics of Long Ties 5

D Life Events and Long Ties 5
D.1 Inter-State Migration . . . 5
D.2 Switching High Schools . . . 5
D.3 High School Closure . . . 5
D.4 Out-of-state College Attendance . . . 6
D.4.1 Data and Methods . . . 7
D.4.2 Results . . . 7
D.5 Self-Reporting as Potential Confounder . . . 8
D.6 Associated Economic Outcomes . . . 8

E References 23

A. Geographically-Aggregated Associations

We use each zip code in the US or Mexico as a unit of analysis and construct a network for each zip code corresponding to the communication patterns of its residents. Fig. S1 illustrates how each zip code network is constructed. It includes all the users residing in a zip code and all their contacts as the nodes and all the ties among them. Fig. S2 shows the histogram of some basic characteristics of the zip code networks in the US, along with a few economic indicators obtained from census. The main approach is to examine the relationship between the overall network structure of each zip code and an array of economic indicators on that zip code provided by the census bureau. In the main text, we present our results on the link between structural diversity, measured as the fraction of long ties, and three important economic outcomes. In particular, the independent variable is the fraction of long ties: the fraction of dotted black edges over all black edges, where dotted pattern indicates a long tie and black color indicates a tie involving at least one user residing in the zip code. The second histogram in second row of Fig. S2 indicates that the majority of edges that are adjacent to a blue node in the zip code are to red nodes outside the zip code. Even a larger majority of long edges that are adjacent to nodes in the zip code are to nodes outside the zip code (histogram not shown). Our secondary analysis examined the strength of long ties which is the sum of edge weights on dotted black edges normalized by the total edge weight on black edges. As explained in the main text, the edge weight incorporates the number of exchanges in either direction.

Figs. S4 and S5 present the relationship of the fraction of long ties and the weighted long ties with zip code outcome variables other the median income presented in the main text. The binned regression models used to generate these plots are the same as one described in *Materials and Methods*. The main conclusion from Fig. S4 is that the residents in zip codes with better outcomes tend to have more long ties. Fig. S4 shows how the zip code outcomes change as the long ties in the zip code network become stronger, conditional on the fraction of long ties. These plots suggest that given a fixed number of long ties in a zip code network, the zip code outcomes tend to be better if the long ties are stronger. This finding points to the opportunity and information value of long ties since the stronger a tie is, the more likely valuable resources and information will be transmitted across the tie.

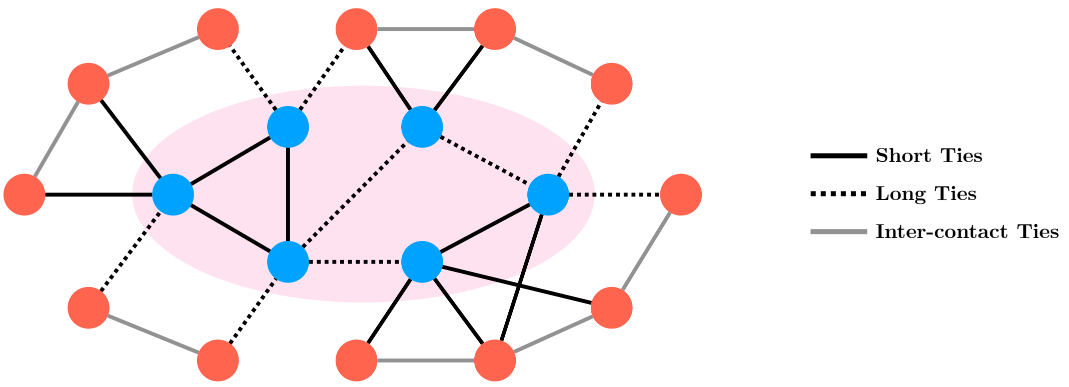

Fig. S1. A schematic representation of a network for a single region (zip code). Blue nodes correspond to individuals residing in the zip code, the purple shaded area indicates the zip code boundary, and the red nodes are contacts who reside in other zip codes. Black edges involve at least one node inside the zip code, and gray edges involve two nodes outside the zip code. Dotted lines correspond to long ties involving at least one node inside the zip code, to a contact either inside or outside the zip code. Our primary analysis considers such long ties represented with a dotted pattern.

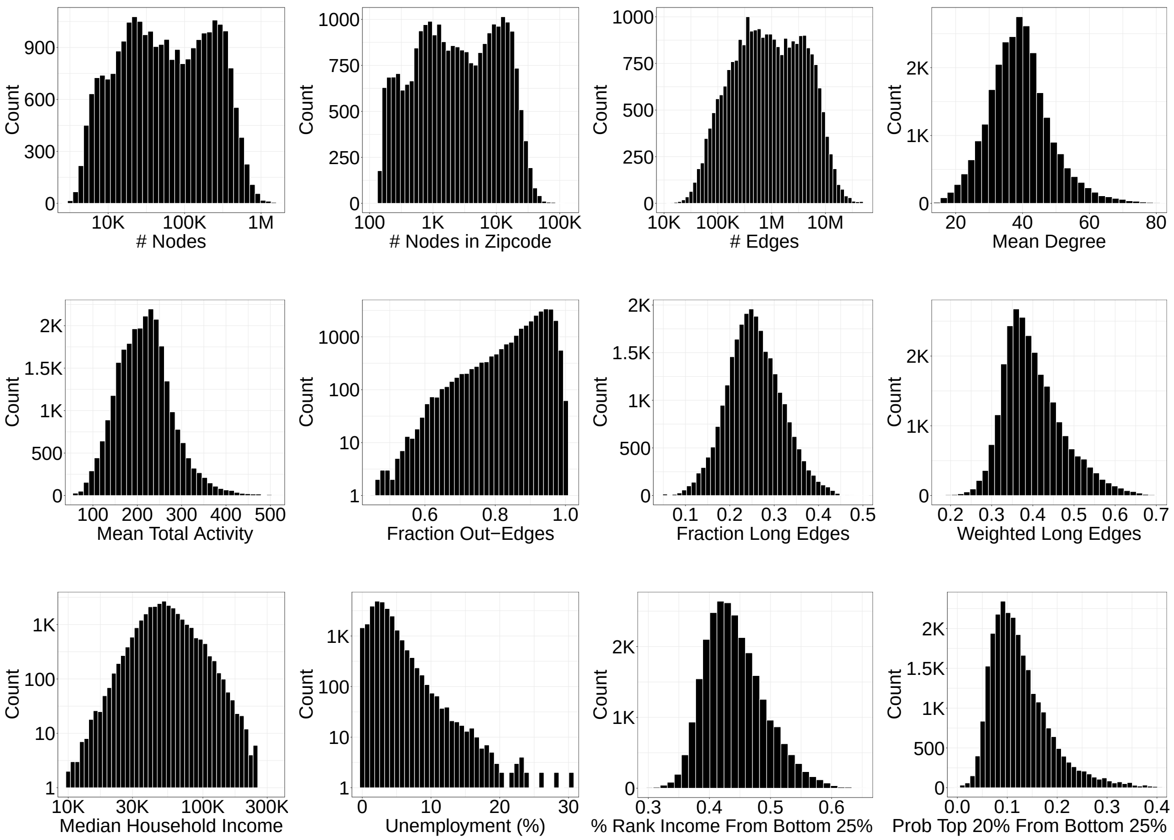

Fig. S2. Histogram of some basic metrics from US zip code networks, along with their household income and unemployment rate from census, and social mobility measures obtained from Atlas of Opportunity project. Mean degree/total activity refers to average degree of/number of comments sent by nodes inside the zip code. Fraction out-edges is the fraction of total edges that originate from a node inside the zip code to one outside the zip code. % rank income from bottom 25% indicates the percentile income rank of a child born in the bottom 25% income group. Similarly, the probability top 20% from bottom 25% refers to the probability that a child born in the bottom 25% income group can make it to the top 20% income group.

B. Individual-level Associations

In the main text, we showed the relationship between (weighted) fraction of long ties and log median family income of residence zip code, number of unique devices and number of donations. Those results were obtained using (generalized full) matching of ego networks per state. Fig. S6 provides further evidence on the this relationship by using three other proxy variables for economic well-being at the individual level: log mean family income of residence zip code (as opposed to the median in the main text), number of unique countries other than Canada, Mexico and Puerto Rico visited since 2013 in trips shorter than 3 weeks and price of main mobile device used to login to Facebook. These results generally confirm the findings in Fig. 2 that higher fraction of long ties or stronger long ties tend to be associated with better economic proxies. The relationship between strength of long ties and mobile phone price is the notable exception as it exhibits largely a null or inverted U-shape behavior with large standard errors. This could be due to either due to measurement errors on phone price or observations on weighted long ties with extremely large values which might behave differently than the rest of the population. The observations with fraction (weighted) of long ties larger than 0.4 account for small fraction of the population as indicated in Fig. S8 and did not appear in the aggregate results due to averaging at the zip code level.

Examining this relationship per state has multiple advantages over a single country-level relationship. First, it provides insights on the existence of heterogeneity of the relationship, if any between the states. Second, it provides a good understanding on the variability of the estimated effect if there is cluster-level dependence between the units. Third, it is computationally tractable as opposed to a single country-level analysis with tens of millions of data points. The remainder of this section explores the robustness of our findings in the main text by employing a different methodology for estimating the marginal effect of (weighted) fraction of long ties on either outcome.

B.1 Average Marginal Effects with Random Forests

As opposed to the matching approach which generates multiple effect sizes, one per a discretized level of long ties, here we estimate a single quantity that captures the average marginal effect of fraction of long ties or weighted long ties on the outcome. In particular when the independent variable is the fraction of long ties, the estimand is the following quantity:

$$\mu_l = E[\frac{\partial f(l_i, l_i^w, C_i)}{\partial l_i}]. \tag{S1}$$

where l_i is user i fraction of long ties as defined in Equation 1, l_i^w is user i weighted long ties as defined in Equation 3, C_i is the set of other covariates, $f(.)$ is a function that maps the covariates to the outcome value and the expectation is taken over the joint distribution of all covariates. The covariates in C_i are the same as those used to obtain the matching results for weighted long ties in the main text: user degree, average degree of user's contacts, daily login activity of the user, average daily login activity of their contacts, average weight of user's edges, age and gender of the user. The quantity in Equation S1 can be interpreted as the average marginal effect of fraction of long ties on the outcome. A similar quantity captures the average marginal effect of weighted long ties on the outcome using the same function $f(.)$:

$$\mu_l^w = E[\frac{\partial f(l_i, l_i^w, C_i)}{\partial l_i^w}]. \tag{S2}$$

The estimation of the marginal effects above can use an approach similar to that used for Accumulated Local Effects (1). First, we train the prediction function $f(.)$ using a random forest and obtain $\hat{f}(.)$. Second in order to estimate the local marginal effect, we divide the variable of interest (either l_i or l_i^w) into many small intervals and compute the difference in $\hat{f}(.)$ prediction along each interval end points. This procedure estimates the local derivative or marginal effect. The estimated average marginal effect is the average of such local marginal effects over the empirical distribution of other observed covariates:

$$\hat{\mu}_l = \frac{1}{n}\sum_{k=1}^{K} \sum_{\{i: l_{k-1} \le l_i < l_k\}} \left[\frac{\hat{f}(l_k, l_i^w, C_i) - \hat{f}(l_{k-1}, l_i^w, C_i)}{l_k - l_{k-1}}\right] \tag{S3}$$

where k denotes the $\mathrm{k^{th}}$ grid interval on l_i or fraction of long ties from l_{k-1} to l_k, K is the total number of intervals and n is the total number of points. The inner sum is the total marginal effect over all the points that fall in the $\mathrm{k^{th}}$ interval. A similar estimator also exists for the average marginal effect of weighted long ties:

$$\hat{\mu}_l^w = \frac{1}{n}\sum_{k=1}^{K} \sum_{\{i: l_{k-1}^w \le l_i^w < l_k^w\}} \left[\frac{\hat{f}(l_i, l_k^w, C_i) - \hat{f}(l_i, l_{k-1}^w, C_i)}{l_k^w - l_{k-1}^w}\right] \tag{S4}$$

Fig. S7 shows the estimated marginal effects of fraction of long ties and weighted long ties on five individual-level outcomes variables: median family income of residence zip code, number of unique

devices used more than once to login to Facebook, number of donations on the Facebook fundraising platform, number of countries visited in potentially troustism trips, and phone price. There is an estimate per each state, obtained solely based on users who live in that state, and an overall estimate which is the average of all state estimates. The marginal effects correspond to 1 percentage point increase (or 0.01) in fraction of or weighted fraction of long ties (l_i and l_i^w). The findings are consistent with the results in Fig. 2 of the main text as the marginal effect of both fraction of long ties and weighted long ties are consistently positive for all five outcome variables suggesting that people with structurally more diverse networks tend to have better outcomes along these variables. Fig. S7 also shows how the marginal effect estimates will change if the model accounts for more covariates. Model 1, represented by blue points, adjusts for the set of covariates C_i mentioned above. Model 2, represented by red points, also adjusts for highest education level (e.g. high school, college, graduate, ...) and median income of home county where user is from. Model 2 provides a basic assessment on the sensitivity of model 1 to potential unobservables. The estimated coefficient remain positive and highly significant in model 2. It should be noted that the standard errors used to construct 95% confidence interval around the overall average marginal effect is cluster robust at the state level since it is the standard error of 51 independent state (including Washington DC) estimates.

C. Demographics of Long Ties

Fig. S8 illustrates the distribution of long ties fraction over individual user ego-networks across various demographic attributes, including gender, age, highest level of education, population density of residence zip code, census region and division. For each attribute, the first plot compares the mean fraction of long ties over different groups and ego-network degree brackets. In contrast to the post-stratified means in Figs. 4A and 4B, per degree bracket means in Fig. S8 are not stratified. The second plot for each demographic attribute compares the conditional distribution of long ties fraction per group, such that the columns sum up to 1. The results suggest that younger, higher educated individuals living in more dense urban areas tend to have more long ties than older, lower educated individuals in less dense rural areas. Furthermore, individuals living in the western states tend to have more structurally diverse networks than the rest of the country.

D. Life Events and Long Ties

D.1 Inter-State Migration

In the main text, we show how inter-state migrants in the US have more long ties than locals. Our analysis involves comparing the post-stratified mean of fraction of long ties between the two groups. Fig. S9 illustrates the distribution of the control variables used in post-stratification (gender, age, home county income) in addition to degree and current state in the US by each group.

D.2 Switching High Schools

Fig. S10 compares the distribution of post-stratification variables in addition to degree, inter-state migration status and current state between the population of users with single or multiple high schools attended.

D.3 High School Closure

The main text describes the data collection around closure of high schools. Our analysis compares users who attended a high school during its closure to those who left the high school immediately before its closure. Fig. 5 compares the stratified mean fraction of long ties across the two high school closure conditions where the strata is defined as the combination of high school and age bracket. This design

effectively compares similar cohorts from the same school who only differ on the period they attended the school by a maximum of 5 years. The age stratification attempts to account for differences in fraction of long ties that might be due to age. However, this natural experiment itself makes it difficult to stratify on a single year since cohorts in the two conditions will be different in age by design which implies some age strata will not include both closure conditions under a fine grained age stratification. Hence, Fig. 5 uses a coarse age stratification with brackets of 4 years. This method leads to many strata which contain both conditions and retains a large fraction of the data, nevertheless it might falsely attribute any age-related variation in fraction of long ties within an age strata to high school closure since those affected by the closure tend to be younger.

In order to fully account for age variation, we use the following two-way linear fixed effect model:

$$Y_i = \beta I_{i,\text{closure}} + \gamma_{S_i} + \lambda_{A_i} + \epsilon_i \tag{S5}$$

where Y_i is the ith subject fraction of long ties in their ego-network, γ_{S_i} is the school fixed effect of the ith subject's school S_i, λ_{A_i} is the age fixed effect for age in integer years, and $I_{i,\text{closure}}$ is an indicator variable that takes a value of 1 if the ith subject attended the school during its closure (i.e. their self-reported end date is within one year of the school closure). In contrast to Fig. 5 which uses stratification, this linear model does not require both conditions to be present within each school–age combination, thus it allows for fine-grained adjustments for age. The third column in Table S1 shows the estimates from this model where standard errors are cluster-robust at the school level. The other columns exclude either one or both fixed effects. The results from this table are consistent with the findings in Fig. 5 in the main text: Facebook users who attended a high school during its closure tend to have more long ties in their networks many years after the event compared to similar cohorts who attended the same school prior its closure.

Table S1. The effect of high school closure on fraction of long ties in the ego-network. The first column regresses fraction of long edges only on an indicator variable with value of 1 when the user has attended the high school during its closure as defined in *Materials and Methods*. The second column also includes school fixed effects. The third column is a two-way fixed effects model with both high school and age fixed effects where age is measured in years. All standard errors are cluster-robust at the school level.

	Fraction of Long Edges		
	(1)	(2)	(3)
During School Closure	0.017***	0.022***	0.012***
	(0.005)	(0.004)	(0.004)
School Fixed Effects	No	Yes	Yes
Age Fixed Effects	No	No	Yes
Observations	33,813	33,813	33,790
R^2	0.001	0.064	0.067
Adjusted R^2	0.001	0.056	0.057
Residual Std. Error	0.299 (df = 33811)	0.290 (df = 33527)	0.290 (df = 33421)
F Statistic	23.308*** (df = 1; 33811)	8.034*** (df = 285; 33527)	6.560*** (df = 368; 33421)

Note: *p<0.1; **p<0.05; ***p<0.01

D.4 Out-of-state College Attendance

In the main text, we discuss how the experience of inter-state migration and switching high schools within a single state is associated with higher fraction of long ties years after the event. In this section, we discuss the experience of another disruption, out-of-state college attendance, and its link to network structure years later. In particular, we examine US-born Facebook users who attended a college outside

their hometown state and compare them with the population of users who attended a college inside their home state. We show that out-of-state college attendance is associated with higher fraction of long ties years later after the college, and this relationship persists if one looks at friends made out of college or account for the inter-state migration effects of out-of-state college attendance.

D.4.1. Data and Methods. We obtain college attendance data on each user based on their self-reported higher education institution. Overall, more than 47% of the active users in our data have attended a college and have optionally reported the name of the higher education institute attended in their Facebook profile. The self-reported information is generally trustworthy, since we have resolved them against known higher education institutions. Our analysis will examine the subset of users aged between 30 and 60 who have attended at least one college, and divide them into two groups of in-state and out-of-state college attendance. Comparing the state where the user attended a college with their hometown state, which is also self-reported as also used and explained in the case of inter-state migration, we can determine whether at least one attended college is outside the user home state. The users with at least one such college constitute our out-of-state population and the rest are considered in-state. In a manner similar to the cases of inter-state migration and switching high schools, we match each individual in our population with their 6 month communication ego network and discard individuals with low levels of activity (degree less than 10 over the 6 month period). The final data consists of 23.5 million users, out of which 15.3 million (65%) attended only colleges within their home state and 8.2 million (35%) attended at least one out-of-state college.

In order to compare users by the location of college attendance, we employ the same approach as explained in the *Materials and Methods*. We compute the post-stratified mean of the fraction of long ties both conditional and unconditional on the degree bin and for each college attendance group. All combinations of gender, age and hometown county income constitute the 90 strata of the analysis. Fig. S11 shows the distribution of these control variables for both groups. An important observation is that the degree and age distributions of in-state and out-state college groups do not have noticeable differences. In contrast, males tend to attend out-of-state college at a higher rate, and users from richer counties are also more likely to attend a college out of their home state. Any difference in fraction of long ties between in-state and out-of-state user groups could partly be attributed to the effect of migration too since users who attended out-of-state college are more likely to permanently move out of their home state, as clearly verified in Fig. S11. However, it is not clear whether one should control for migration status by including it as a stratum, since the two mechanisms of out-of-state college attendance and inter-state migration only partly overlap and even if they were not distinct, it is difficult to separate them. Nevertheless in our secondary analysis, we include the binary inter-state migration status as another stratum in the post-stratification, which leads to 180 total strata. We will present results comparing the two groups controlling for either stratum definition.

D.4.2. Results. Overall, we find that the out-of-state group has about 9% more long ties relative to in-state users among all ties (34.1% vs. 31.3% in absolute terms with difference-in-means $p < 10^{-10}$ using cluster-robust standard errors at the county level) and 10.0% more long ties relative to in-state users among ties outside college (34.3% vs. 31.3% in absolute terms with $p < 10^{-10}$ using county-level cluster-robust standard errors). Fig. S12 further conditions on user degree and compares the fraction of long ties between users by location of college attendance, using two different stratum definition. The left plot compares the overall fraction of long ties. Similar to the case of inter-state migration, the higher fraction of long ties might be due to exposure to college friends from a different state, rather than an acquired skill. However, the inset plot shows that users with out-of-state college attendance have more long ties even among contacts who did not attend any of their colleges. This suggests that their current higher likelihood to have long ties is not directly due to connections with diverse college friends in the past. Out-of-state users are much more likely to be inter-state migrants as shown in

Fig. S11, hence the right plot attempts to control for the migration status, even though this might underestimate the effect of out-of-state college attendance. Accounting for migration status reduces the absolute difference between the two groups (marginal over degree distribution) from 2.8% to 1.4%, however users with the experience of out-of-state college have consistently more (statistically significant) long ties in all degree bins. This suggests the higher structural diversity in the out-of-state group is not solely due to inter-state migration.

D.5 Self-Reporting as Potential Confounder

The differences on the fraction of long ties for those with and without the events we study might might be partially attributable to other differences between the two populations. This is particularly true since assignment to either group (migrant vs. local or multiple high schools vs. single high school) is based on self-reported information on user's profile. For example, it might be that users who tend to reveal more information about themselves (e.g., both of their high schools rather than only the last one) also happen to hold more long ties. We cannot completely rule out this concern; however, we can compare the two populations by how much information they share in their Facebook profiles and examine whether the differences in the fraction of long ties is driven by the differences in propensity to self-report personal information. In this section, we compare migrants versus non-migrants, out-of-state versus in-state college attendees and multiple high schools versus single high school students by how many of the following fields are completed on their Facebook profile: relationship status, current city, employer information and mobile phone number. For each user, we compute the count of such fields (out of 4) reported in their profile and compare the fraction of long ties between the groups with different number of disclosed fields. In terms of overall propensity to disclose information, we observe that migrants, multiple high school attendees and out-of-state college students on average disclose 4.0%, 5.8% and 1.8% more fields relative to non-migrants, single high school attendees and in-state college students respectively.

But are the differences in fraction of long ties due to differences in propensity of self-reporting personal information? We try to address this question by comparing the post-stratified mean of long ties between the groups conditioned on the the number of these fields reported in the Facebook profile (minimum of 0 and maximum of 4). Fig. S13 compares the fraction of long ties between the two groups in each type of life event (inter-state migration, switching high schools, and attending out-of-state college) across different number of disclosed fields within each group. These results do not differ much compared to the ones obtained when post-stratification is conducted on the whole group as reported in the text and shown in Figs. 4A, 4B and S12 rather than subgroups with a fixed number of disclosed fields. Furthermore, we observe that migrants, multiple high school attendees and out-of-state college students consistently have more long ties than non-migrants, single high school attendees and in-state college students no matter which sub-groups based on the number of disclosed fields we compare. Thus, we believe the differences in the fraction of long ties are not confounded due to the propensity of self-reporting personal information, at least given the analysis described above.

D.6 Associated Economic Outcomes

The main results on determinants of long ties show that three major disruptive events are associated with higher fraction of long ties later on in life. But they don't address whether those events lead to better outcomes too, specially since we showed the link between long ties and outcomes at an aggregate scale in the first part of the article. Establishing the link between these events and outcomes is challenging due to lack of important outcome data at the individual level. For illustrative purposes and to provide preliminary evidence of this link, we focus on two outcomes: the mean household income of current county and the mobile phone price. The first outcome allows to answer whether people with the experience of those events live in richer counties. The current county is determined using Facebook

data based on location signals as explained previously and mean household income of US counties is obtained from 2018 ACS census. Mobile phone model, matched against estimated prices, is available from the user connection logs.

Fig. S14 shows the mean difference in the outcomes of migrant and local populations (positive values indicate migrants live in richer counties or have more expensive mobile phones) broken down by the racial composition of the hometown county where the user grew up. Hometown county is determined based on the self-reported hometown city in the user's profile. Both plots indicate that inter-state migration is increasingly associated with better outcomes in predominantly white counties, consistent with previous findings (2, 3). The upper limit of racial composition brackets approximately correspond to 20th, 40th, 60th, 75th, 90th and 100th percentiles of white population in US counties as indicated in census data. Figs. S15 and S16 show similar results comparing the population of multiple high schools versus single high school attendees and out-state versus in-state college students. In particular considering the case of switching high schools, users who attended multiple high schools have more expensive phones than those who attended a single high school regardless of the racial composition of their home town counties. However, only those users who grew up in predominantly white counties (with white population beyond the 75th percentile of all US counties) currently live in richer counties.

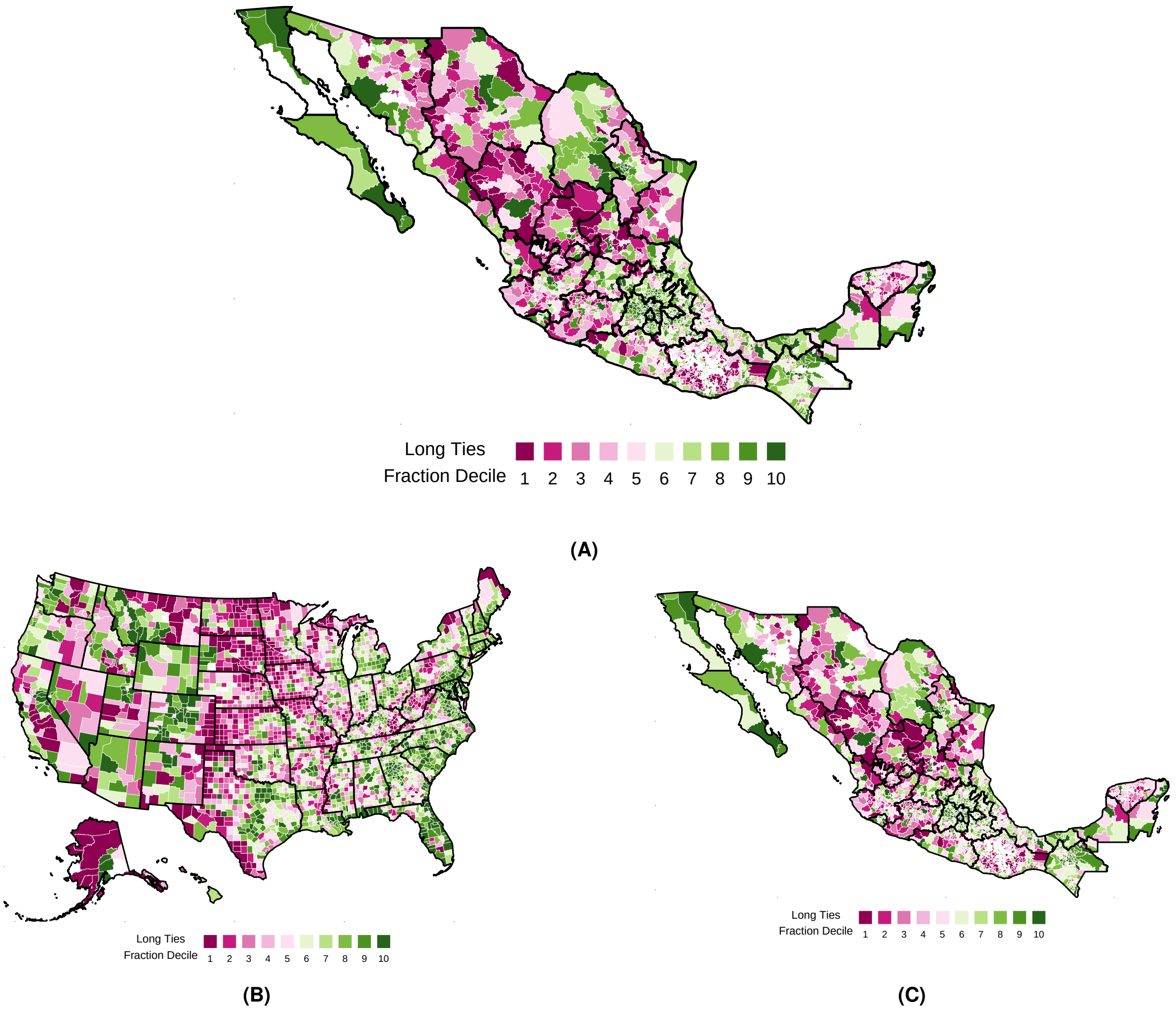

Fig. S3. (A) Proportion of Facebook ties that are long, measured across across municipalities in Mexico, and after controlling for the average number of Facebook contacts. The adjusted (residualized) fraction of long ties across administrative units in each country is binned into deciles, with lower values shown in purple and higher values shown in green. Administrative units with less than 200 Facebook users sufficiently active over the measurement period or with less than 500 individuals are shown in white. (B and C) Proportion of Facebook ties that are long, computing using the public release of geographically-aggregated data associated with this paper. These differ from panel A and Fig. 1A in (a) being computed over a different period (180 days ending in November 2021) and (b) including a somewhat different subset of administrative units, both due to (a) and because the public data release uses a threshold of 150 sufficiently active users.

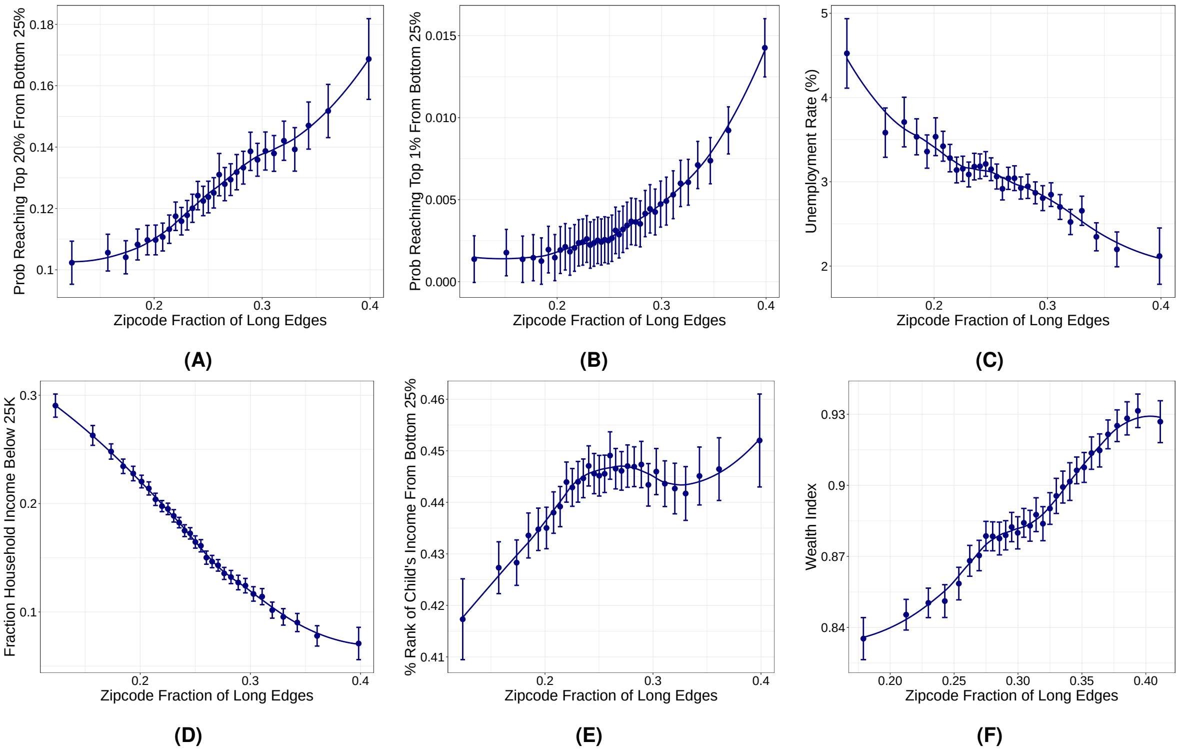

Fig. S4. Fraction of long ties and geographically aggregated economic outcomes in US **(A-D)** and Mexican **(E)** zip codes. The binned regression plots are generated according to model (6) in *Materials and Methods*, which adjusts for the (binned) size of the zip code, its racial composition, and average degree in the network. Solid lines and bars correspond to local smoothers of second degree and 95% confidence intervals in each bin respectively. Standard errors are cluster robust at the county level.

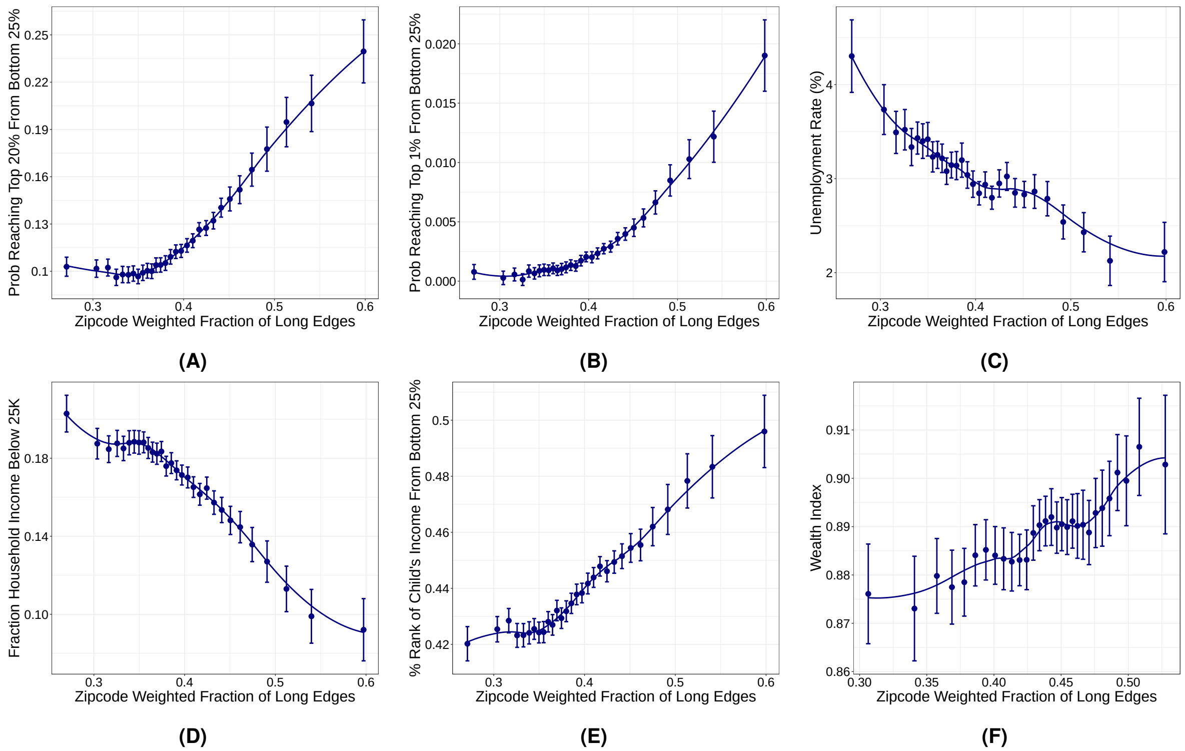

Fig. S5. Interaction-count weighted fraction of long ties and geographically aggregated economic outcomes in US **(A-D)** and Mexican **(E)** zip codes. The binned regression plots are generated according to model (7) in *Materials and Methods*, which adjusts for the (binned) size of the zip code, its racial composition, average degree in the network, and unweighted fraction of long ties. Solid lines and bars correspond to local smoothers of second degree and 95% confidence intervals respectively constructed using cluster robust standard errors at the county level.

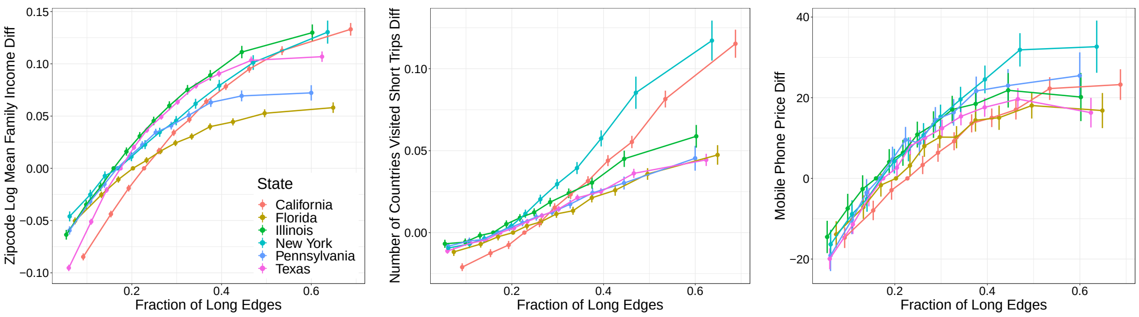

(A)

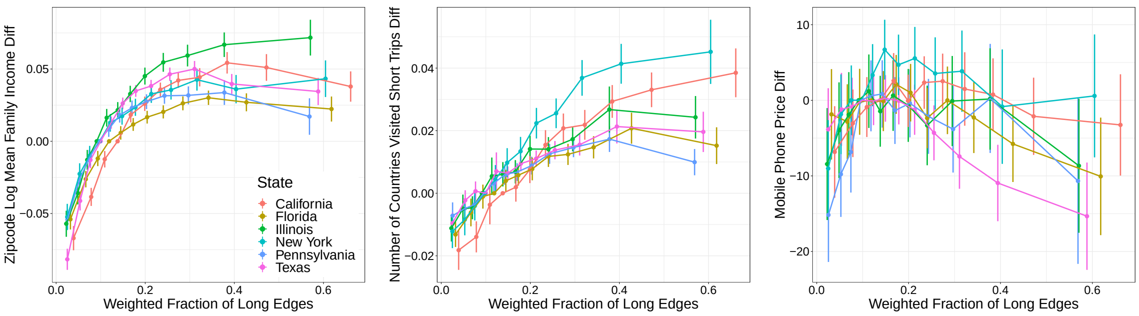

(B)

Fig. S6. Fraction of long ties **(top row)** and the tie-strength weighted fraction of long ties **(bottom row)** in ego networks and individual level variables that are positively correlated with socio-economic status for users who reside in the top 6 most populated states in the US. The plots are generated based on the procedure in *Materials and Methods* which matches individuals based on their degree, average degree of their contacts, their daily login activity, average daily login activity of their contacts, their age and gender for the top row. The matching for the bottom row also incorporates the fraction of long ties and average edge weight in addition to the parameters mentioned before. Bars correspond to 95% confidence intervals. Values in the y-axis correspond to the difference with the baseline represented with a value of zero.

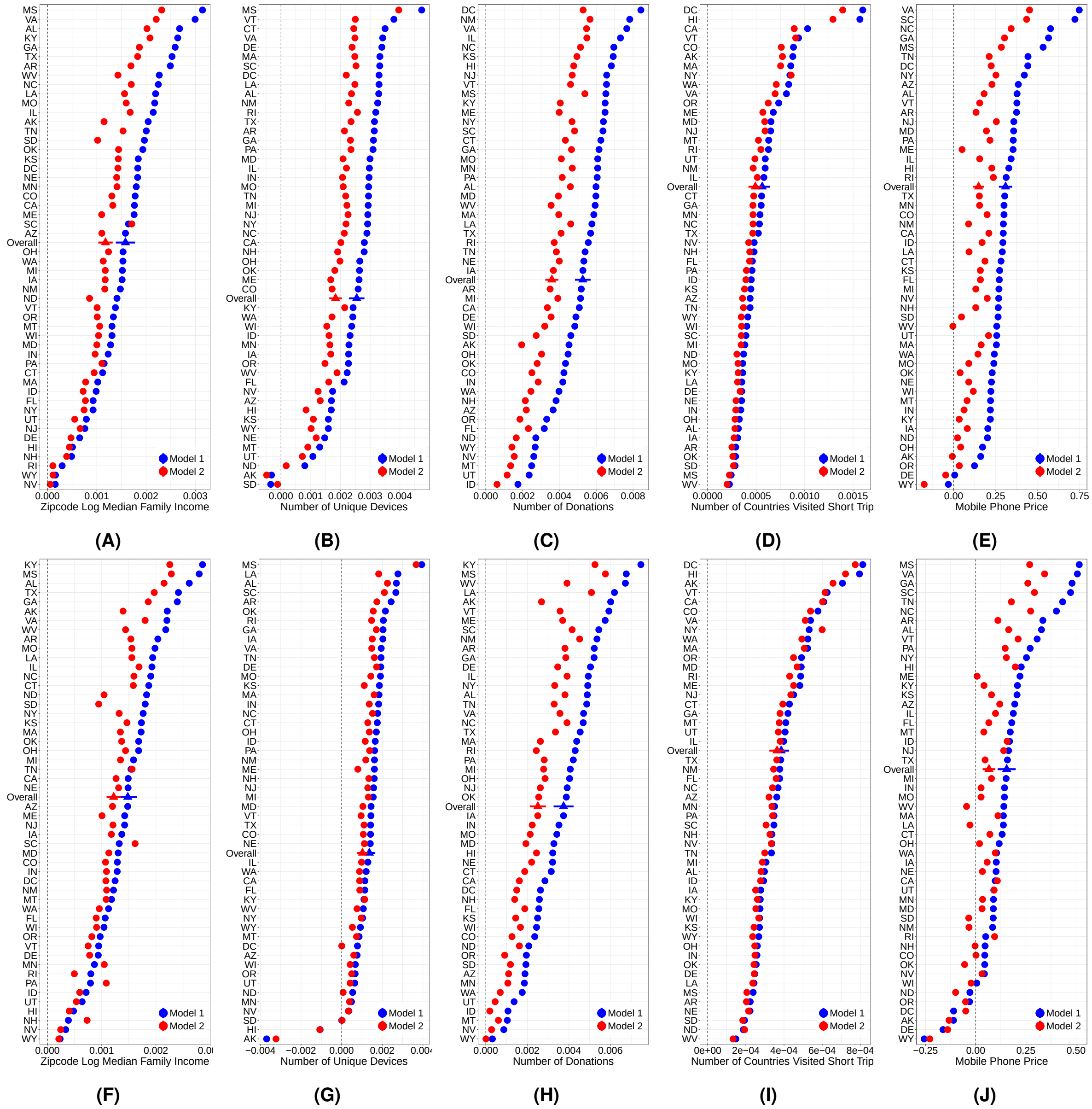

Fig. S7. The average marginal effect of fraction of long ties (**Top row**) and weighted long ties (**Bottom row**) on median family income of residence zip code (**First column**), number of unique devices used to login to Facebook (**Second column**), number of fundraising donations (**Third column**), number of countries visited in short trips (**Fourth column**), and phone price (**Fifth column**). Model 1 accounts for the baseline covariates discussed in the main text. Model 2 also accounts for education level and home county income. Each dot corresponds to the estimated marginal effect of 1% (0.01) increase in fraction or weighted long ties for residents of a single state. The triangle corresponds to the overall marginal effect as the average of all state-level estimates. There is no estimated standard error for state-level estimates, but the standard error of the mean can be used for the overall estimate. The bars corresponds to 95% confidence interval for the overall estimate that is cluster robust at the state level.

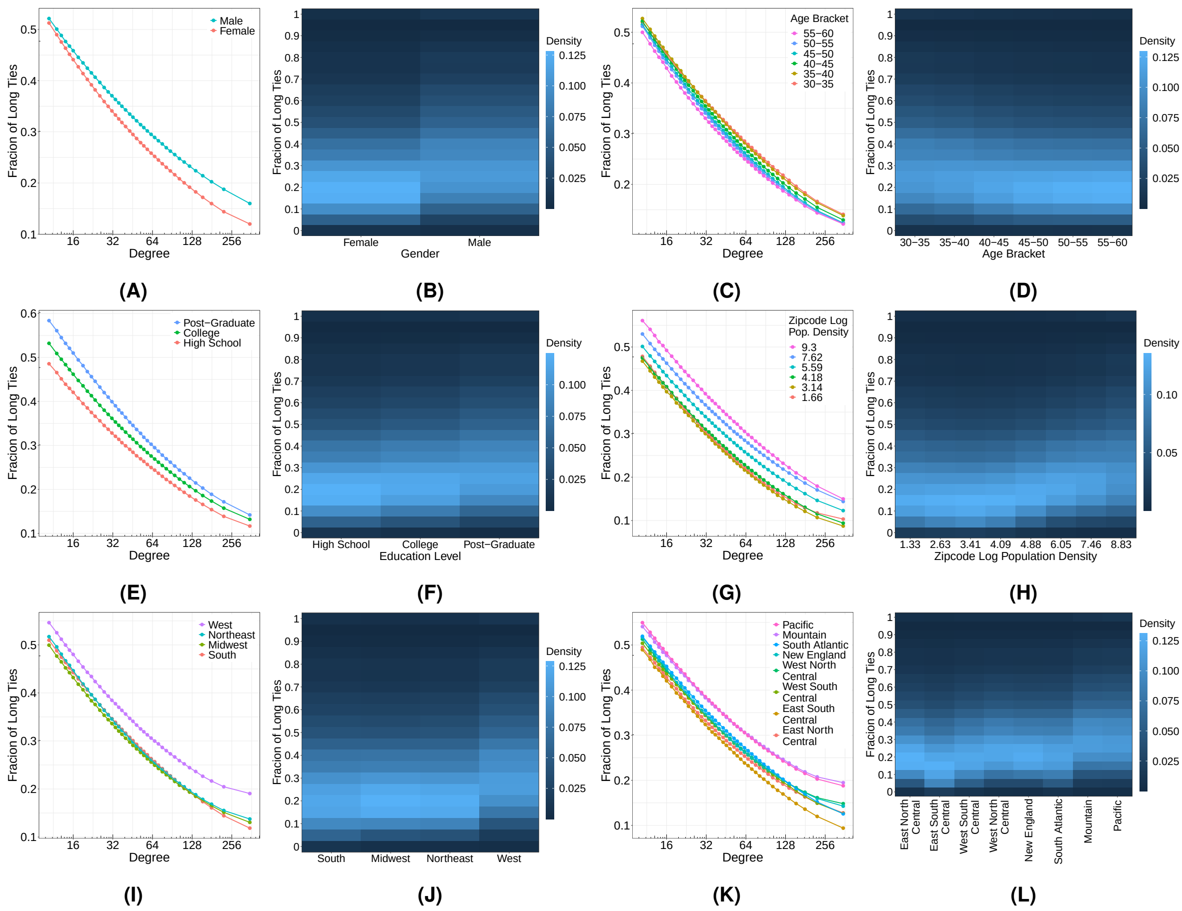

Fig. S8. The distribution of long ties across several demographic variables, including gender, age, education, population density of residence zipcode, residence census region and census division. Panels A, C, E, G, I and K compare the average fraction of long ties versus the ego network degree within each demographic category. Panels B, D, F, H, J, L show the conditional density of fraction of long ties within each level of the category.

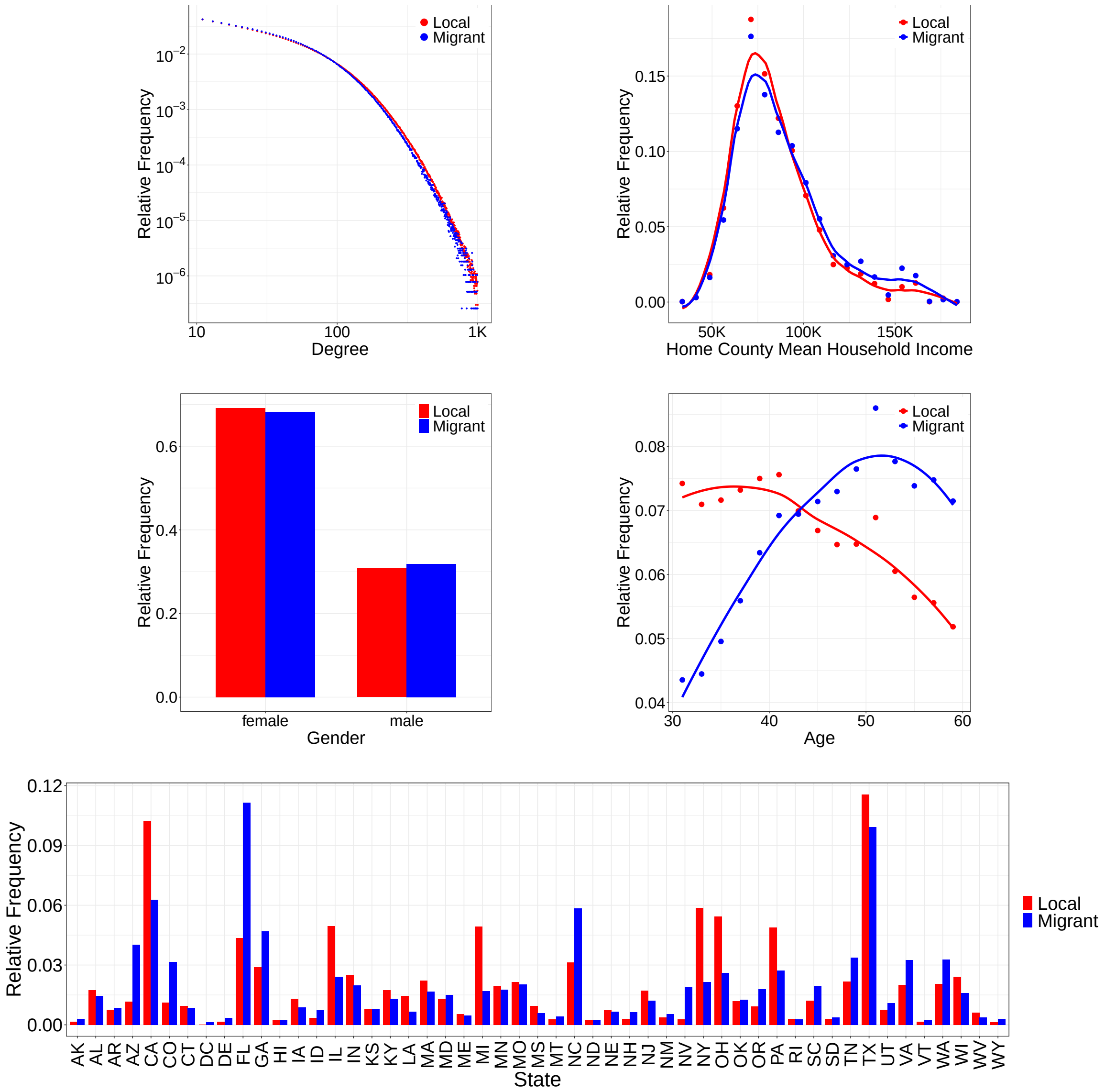

Fig. S9. The relative histogram of degree, hometown county income, gender, age and current state by migration status. The frequencies sum to 1 within each migration group. The degree distribution is shown with a log-log scale. The solid line in the age and income distributions correspond to a LOESS smoother.

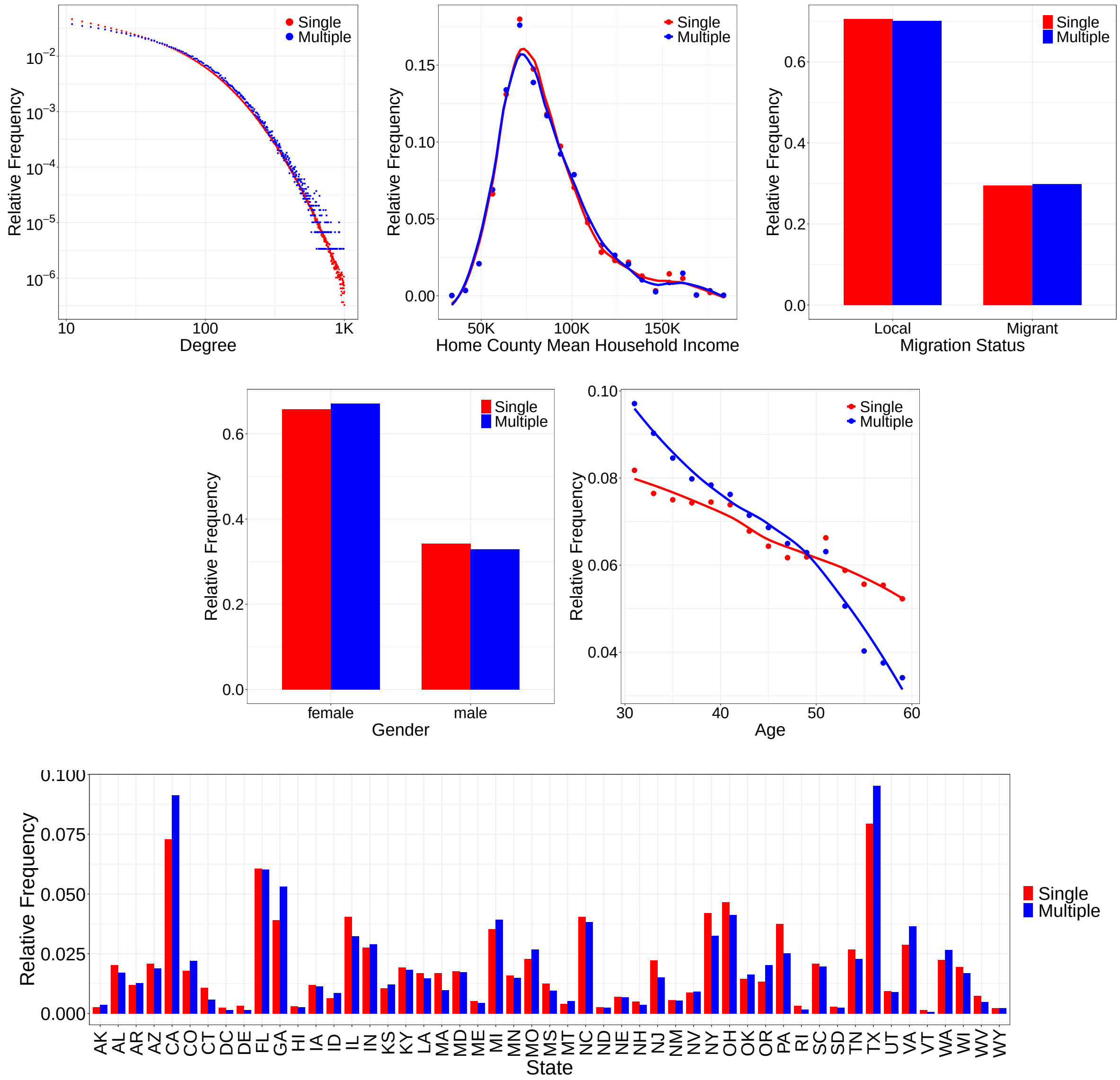

Fig. S10. The relative histogram of degree, hometown county income, migration status, gender, age and current state by number of high schools attended. The frequencies sum to 1 within each high school attendance group. The solid line in the age and income distributions correspond to a LOESS smoother.

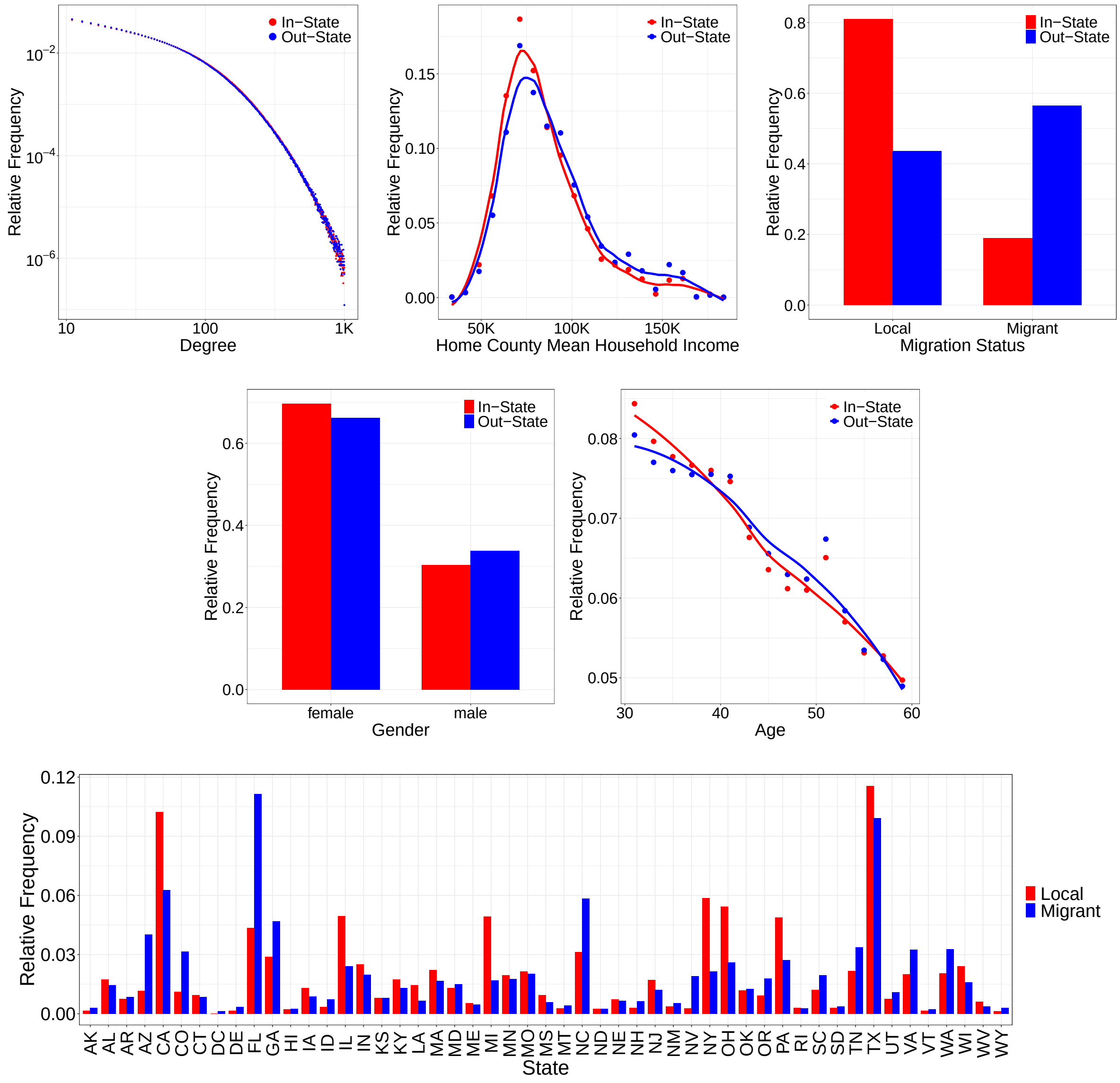

Fig. S11. The relative histogram of degree, hometown county income, migration status, gender, age and current state by location of college attendance. The frequencies sum to 1 within each college attendance group. The solid line in the age and income distributions correspond to a LOESS smoother.

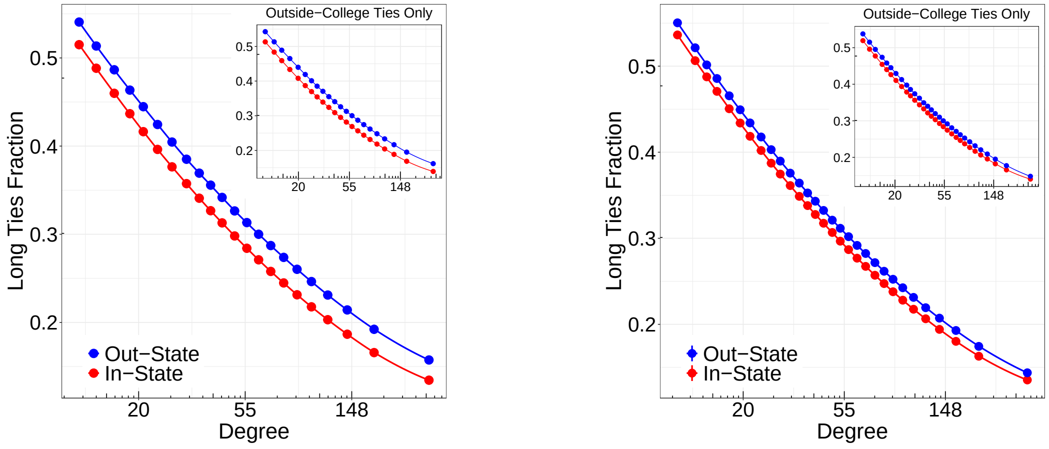

(A) **(B)**

Fig. S12. The stratified fraction of long ties over all ties and within the ties who did not attend the same high college as the user (inset) by degree and location of college attendance (**left**). The combination of gender, age and hometown county income bins constitute the stratification strata for the left and middle plots. The **right** plot also controls for the binary inter-state migration status by including it in the strata definition.

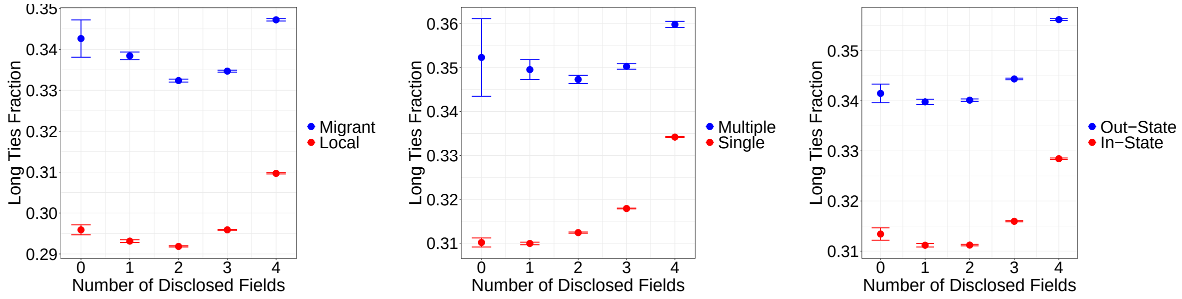

Fig. S13. The stratified fraction of long ties over all ties broken down by the number of fields disclosed in the user profile. The combination of degree, gender, age and hometown county income bins constitute the stratification strata for all plots. Bars correspond to 95% confidence intervals.

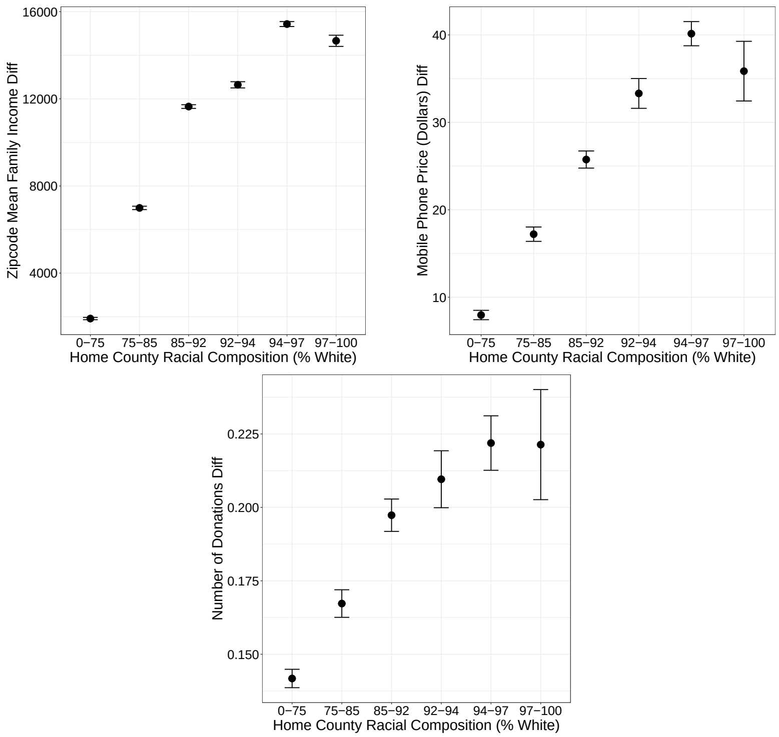

Fig. S14. The difference in current zip code mean family income (**left**), user mobile phone price (**middle**) and number of donations to fundraisers (**right**) of migrants over locals (migrant - local). The differences are stratified in both plots by strata defined as the combination of gender, age and hometown county income bins. Bars correspond to 95% confidence intervals.

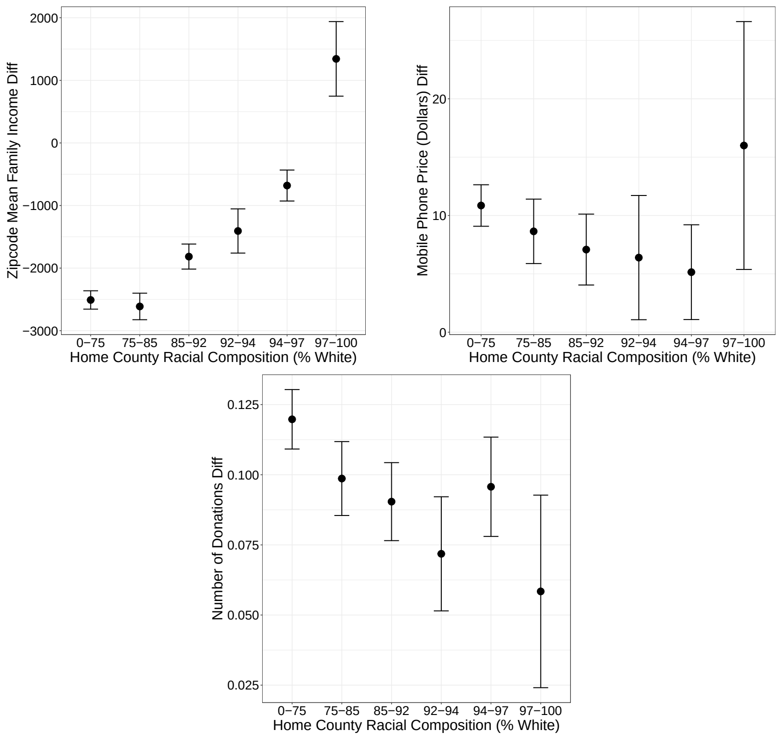

Fig. S15. The difference in current zip code mean family income (**left**), user mobile phone price (**middle**) and number of donations to fundraisers (**right**) of multiple over single high school attendance groups (multiple - single). The differences are stratified in both plots by strata defined as the combination of gender, age and hometown county income bins. Bars correspond to 95% confidence intervals.

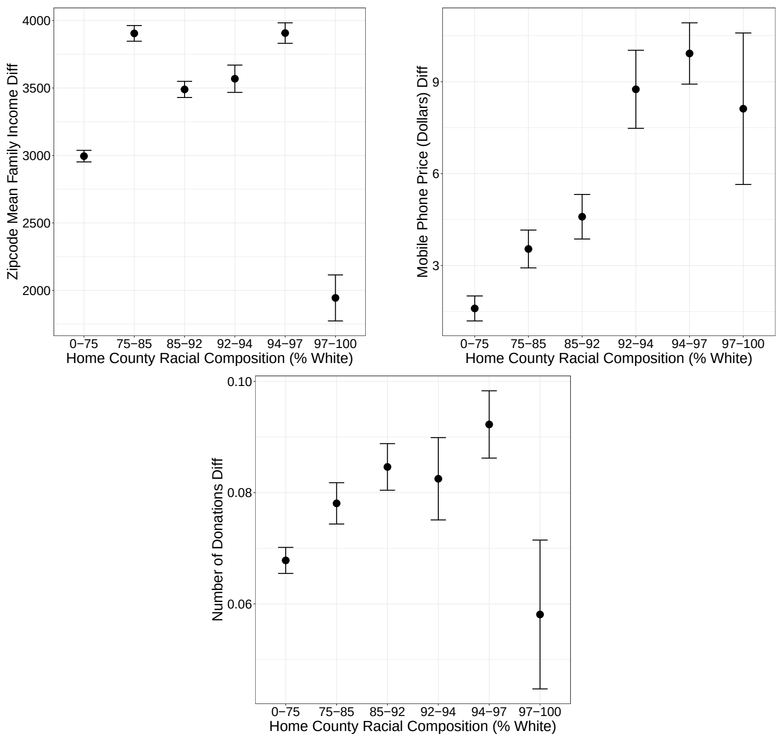

Fig. S16. The difference in current county mean household income (**left**), user mobile phone price (**middle**) and number of donations to fundraisers (**right**) of out-state over in-state college attendance groups (out-state - in-state). The differences are stratified in both plots by strata defined as the combination of gender, age and hometown county income bins. Bars correspond to 95% confidence intervals.

E. References

1. DW Apley, J Zhu, Visualizing the effects of predictor variables in black box supervised learning models. *J. Royal Stat. Soc. Ser. B* **82**, 1059–1086 (2020).
2. R Chetty, N Hendren, P Kline, E Saez, Where is the land of opportunity? The geography of intergenerational mobility in the United States. *The Q. J. Econ.* **129**, 1553–1623 (2014).
3. R Chetty, JN Friedman, N Hendren, MR Jones, SR Porter, The opportunity atlas: Mapping the childhood roots of social mobility, (National Bureau of Economic Research), Working Paper 25147 (2018).
